# High-Density Genetic Map Construction and Identification of QTLs Controlling Oleic and Linoleic Acid in Peanut using SLAF-seq and SSRs

**DOI:** 10.1038/s41598-018-23873-7

**Published:** 2018-04-03

**Authors:** X. H. Hu, S. Z. Zhang, H. R. Miao, F. G. Cui, Y. Shen, W. Q. Yang, T. T. Xu, N. Chen, X. Y. Chi, Z. M. Zhang, J. Chen

**Affiliations:** 1 Shandong Peanut Research Institute, Qingdao, 266100 P.R. China; 2 Institute of Industrial Crops, Jiangsu Academy of Agricultural Sciences, Nanjing, 210014 P.R. China

## Abstract

The cultivated peanut, *A. hypogaea* L., is an important oil and food crop globally.High-density genetic linkage mapping is a valuable and effective method for exploring complex quantitative traits. In this context, a recombinant inbred line (RIL) of 146 lines was developed by crossing Huayu28 and P76. We developed 433,679 high-quality SLAFs, of which 29,075 were polymorphic. 4,817 SLAFs were encoded and grouped into different segregation patterns. A high-resolution genetic map containing 2,334 markers (68 SSRs and 2,266 SNPs) on 20 linkage groups (LGs) spanning 2586.37 cM was constructed for peanut. The average distance between adjacent markers was 2.25 cM. Based on phenotyping in seven environments, QTLs for oleic acid (C18:1), linoleic acid (C18:2) and the ratio of oleic acid to linoleic acid (O/L) were identified and positioned on linkage groups A03, A04, A09, B09 and B10. Marker2575339 and Marker2379598 in B09 were associated with C18:1, C18:2 and O/L in seven environments, Marker4391589 and Marker4463600 in A09 were associated with C18:1, C18:2 and O/L in six environments. This map exhibits high resolution and accuracy, which will facilitate QTL discovery for essential agronomic traits in peanut.

## Introduction

The cultivated peanut (*A. hypogaea* L.) is an allotetraploid (2n = 4× = 40) legume that is mostly grown in semi-arid tropic regions in the world as an important oil and food crop. During 2010–2014, the global average production was 42.27 million tons from an area of 25.27 million ha^1^. A genetic map constructed from a population segregation for a trait of interest is required for QTL (quantitative trait loci) identification. Currently, genetic linkage maps commonly include restriction fragment length polymorphism (RFLP), amplified fragment length polymorphism (AFLP), simple sequence repeat (SSR), and single-nucleotide polymorphism (SNP) markers. A genetic map, especially a high-density genetic map, provides an important foundation for QTL mapping and anchoring sequence scaffolds, and the utility of genetic linkage maps depends on the types and numbers of markers used. Since 2001, 27 previously tetraploid peanut linkage maps were published, 23 of which were cultivated peanut species^2–15^, four of which were from crosses of cultivated and synthetic tetraploid peanut species^16–19^. There were four linkage maps which had more than 1,000 markers including SSR, transposon and SNP. Using the F_2_ population from *A. hypogaea* ‘Satonoka’ and *A. hypogaea* ‘Kintoki’, Shirasawa *et al*. (2012) constructed a high-density genetic linkage map which had 1,114 markers and spanned 2,166.4 cM^9^. Huang *et al*. (2016) constructed a high-density genetic linkage map of cultivated peanut which had 1,219 markers including 1,175 SSR and 42 transposon polymorphic markers, and spanned 2,038.75 cM^15^. Zhou *et al*. (2014) constructed one linkage map which was comprised of 1,685 marker loci, including 1,621 SNP and 64 SSR markers. The map displayed a distribution of the markers into 20 linkage groups (LGs), spanning a distance of 1, 446.7 cM^10^. Shirasawa *et al*. (2013) constructed a genetic linkage map of cultivated peanut with 1,469 markers^19^.

SNPs are the most abundant and stable form of genetic variation in most genomes and have become the marker type of choice in many genetic studies. Recently, SLAF-seq (specific length amplified fragment sequencing), a high-resolution strategy, has been developed for large-scale de novo SNP discovery^20^. Combining NGS, SLAF-seq is a very time- and cost-effective method. The efficiency of SLAF-seq was tested on data from rice^21^ and soybean^22^. This approach has been successfully applied to high-density genetic map construction, QTL analysis and mapping genes for many plants and animals. Since 2013, more than 30 high-density genetic maps have been constructed using SLAF markers, including SLAF-seq map for sesame (*Sesamum indicum*)^23^, kiwifruit (*Actinidia chinensis*)^24^, soybean (*Glycine max*)^25^, mei (*Prunus mume*)^26^, cucumber (*Cucumis sativus*)^27–29^, orchard grass (*Dactylis* spp.)^30^, and red sage (*Salvia miltiorrhiza*)^31^. This approach was also used to detect QTL for the isoflavone content of soybean^32^, growth-related QTL of the Chinese mitten crab *Eriocheir sinensis*^33^, fruit-related QTL of cucumber^31^, flowering time QTLs in orchard grass^29^ and adzuki bean (*Vigna angularis*)^34^. Guo *et al*. used SLAF markers and the BSA (bulked segregant analysis) method to discover QTLs controlling CMV (cucumber mosaic virus) resistance in pepper (*Capsicum frutescens*), and then identified the gene CA02g19570 as a possible candidate gene for resistance to CMV in pepper^35^. These results show that SLAF sequencing is a powerful high-throughput technique for the efficient development of a large number of polymorphic markers in a short time and is effective for linkage map construction and QTL analysis.

In this study, SLAF-seq was used for the rapid discovery of SNPs in the RIL population. Subsequently, we constructed a high-density genetic map of *A. hypogaea* L., which contained 2,334 markers (68 SSRs and 2,266 SNPs) on the 20 linkage groups and spanned 2,586.37 cM, with an average distance of 2.25 cM between adjacent markers. QTLs for oleic acid (C18:1), linoleic acid (C18:2) and the ratio of oleic acid to linoleic acid (O/L) were analysed based on phenotyping in seven environments. This map exhibited high resolution and accuracy and is the first map based on SLAF-seq in peanut. It provides a new method of constructing a peanut genetic map whereby SLAF-seq is applied to peanut. It will facilitate the identification of genes and QTLs underlying essential agronomic traits in peanut.

## Results

### High-throughput SLAF sequencing and genotyping

After SLAF library construction and Illumina sequencing, a total of 64.2 Gb of data containing 322.29 M reads were obtained. The average GC (guanine-cytosine) content was 42.91%, and Q30 ratio (bases with a quality score of 30, indicating a 1% chance of an error and thus 99% confidence) was 87.00%. In the maternal inbred line (Huayu28), the number of reads produced for 398,870 SLAFs was 12,310,352, and the average coverage for each SLAF marker was 21.81-fold. In the paternal line (P76), 11,979,609 reads and 404,089 SLAFs were generated, with an average coverage of 20.68-fold for each SLAF. For the analysis of the RIL mapping population, an average of 2,041,121 reads were generated for the development of 262,928 SLAF markers for each line, and the average coverage was 5.53-fold (Table 1).

**Table 1:** Summary of marker depths.

Samples	SLAF numbers	Total depth (×)	Average Depth (×)
Huayu28	398,870	8,698,059	21.81
P76	404,089	8,355,690	20.68
RILs	262,928	1,455,227	5.53

**Figure 1:**
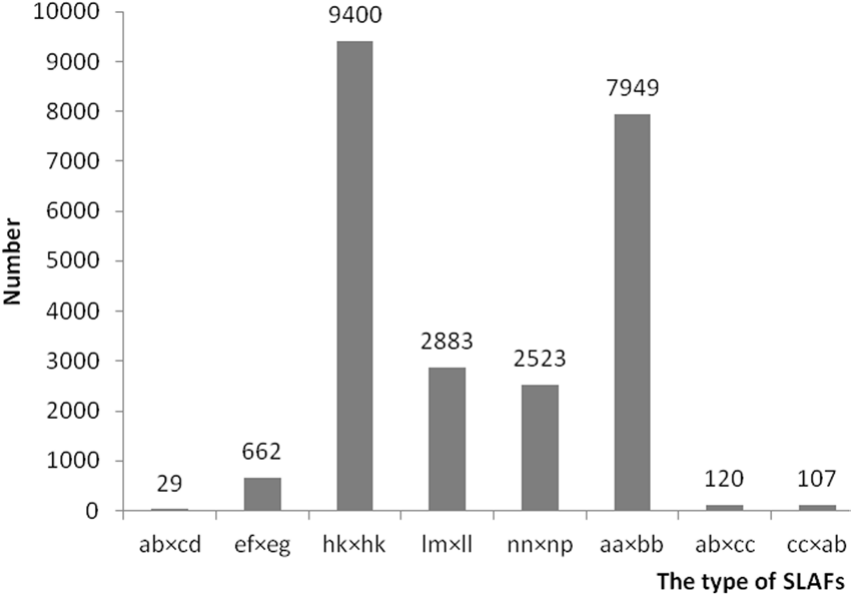
Genotype distribution of SLAF markers.

After correcting or discarding low-depth SLAF tags, 433,679 SLAFs were identified, among which 29,075 were polymorphic with a polymorphism rate of 6.70% (Supplementary Dataset 1). The parental lines were given with different alphabets than genotypes to determine segregation patterns, and 23,673 from the 29,075 polymorphic SLAFs were successfully encoded and grouped into eight segregation patterns (ab × cd, ef × eg, hk × hk, lm × ll, nn × np, aa × bb, ab × cc and cc × ab) following a genotype encoding rule (Fig. 1). Since the two parents (Huayu28 and P76) are homozygous inbred lines with genotypes of aa and bb, only the 7,949 markers that fell into the aa × bb segregation pattern were used in linkage analysis.

### Genetic linkage map

Comparing with the reference genome (http://peanutbase.org/home), HighMap assigned 2,334 of 2,384 markers to 20 LGs of peanut including 2,266 SNPs and 68 SSRs. The total genetic length of the molecular linkage map was 2,586.37 cM in twenty linkage groups with a mean marker distance of 2.25 cM between adjacent markers (Figure S1; Table 2 and Supplementary Dataset 2). The number of markers in each LG ranged from 13 to 369, with an average of 116.7 markers per LG. The LGs ranged from 68.79 to 191.24 cM in length, with average inter-marker distances of 0.3–6.09 cM, and nine linkage groups contained over 100 marker loci. A09 was the shortest, with 156 loci spanning 68.79 cM, while B04 was the longest group, with 240 loci spanning 191.24 cM. B01 contained the fewest markers with 13 loci, whereas B05 had the highest density with 369 loci.

**Table 2:** Description of basic characteristics of twenty linkage groups.

Chr ID	Total markers	SSR Number	Total distance (cM)	Average distance (cM)	Max inter-marker distance (cM)	No. of distorted markers	Frequency of distorted markers	Inter-marker distances ≤5
Aradu.A01	168	6	116	0.69	10.69	0	0.00%	97.01%
Aradu.A02	124	2	148.89	1.21	16.44	10	8.06%	93.50%
Aradu.A03	36	8	130.01	3.71	12.44	9	25.00%	74.29%
Aradu.A04	135	5	120.69	0.9	22.42	129	95.56%	96.27%
Aradu.A05	129	1	88.96	0.69	9.07	0	0.00%	96.09%
Aradu.A06	54	2	170.85	3.22	18.77	9	16.67%	75.47%
Aradu.A07	86	4	176.05	2.07	21.36	23	26.74%	90.59%
Aradu.A08	53	6	160.58	3	12.41	17	32.08%	75.00%
Aradu.A09	156	2	68.79	0.44	9.76	0	0.00%	96.13%
Aradu.A10	22	2	112.51	5.36	12.04	0	0.00%	57.14%
Araip.B01	13	1	73.05	6.09	12.54	0	0.00%	41.67%
Araip.B02	29	1	98.53	3.52	26.24	9	31.03%	78.57%
Araip.B03	240	2	119.45	0.5	12.88	217	90.42%	97.91%
Araip.B04	240	4	191.24	0.8	10.14	1	0.42%	94.56%
Araip.B05	369	5	141.04	0.38	6.60	271	73.44%	99.73%
Araip.B06	74	4	138.32	1.89	18.91	1	1.35%	89.04%
Araip.B07	39	4	139.42	3.67	19.00	2	5.13%	78.95%
Araip.B08	270	2	81.05	0.30	18.07	0	0.00%	99.26%
Araip.B09	40	4	128.97	3.31	19.7	1	2.50%	76.92%
Araip.B10	57	3	181.97	3.25	23.08	1	1.75%	80.36%
Total	2334	68	2586.37	—	—	700	—	—
Mean	—	—	—	2.25	15.63	—	29.99%	—

In the map, 700 (29.99%) markers showed a skewed segregation pattern *(p* < 0.05; Table 2). The segregation distortion markers were distributed in 14 LGs. There were no segregation distorted markers in A01, A05, A09, A10, B01, B08. The ratio of segregation distorted markers in A02, B04, B06, B07, B08, B09 and B10 were lower than 8.06%. The ratios of segregation distorted markers were extremely high in A04, B03 and B05, at 95.56%, 90.42% and 73.44%, respectively.

The SSR markers were distributed across 20 LGs (Figure S1; Table 2, Supplementary Dataset 2 and Supplementary Dataset 3). 15 SSR markers were mapped to the same linkage group by comparing our map and other previous maps (Huang *et al*., 2016 and Peanutbase; Supplementary Dataset 3). For example, GM1992 and IPAHM288, located in A01 and A03 of those previous maps, were grouped in A01 and A03. 9 SSR markers showed different results to those previous maps. IPAHM_356, GM2259, and IPAHM_82, located in A05 and A09 of Peanutbase, respectively, were grouped in A01, A06 and B10 in this study. Twelve SSR markers showed confused positions; for example, TC13E05 was mapped to A02 in this study, while it was located in B02 of Peanutbase. Ah1TC6H03, IPAHM_659 and Ah1TC5D06 showed similar results.

### Validation of the SNP-based genetic map

The quality of this peanut genetic map was evaluated by the haplotype and heat maps. The haplotype maps, which reflect the double exchange of the population, were developed for parental controls and 146 RILs using 2,334 markers (Additional file S1). Most of the recombination blocks were distinctly defined. The heat maps directly reflected recombination relationships among markers in each LG (Additional file S2). Each cell represented a recombination rate between two adjacent markers, the level of which was visualized by different colors ranging from yellow to purple (yellow indicated a lower recombination rate; purple indicated a higher rate). Heat maps indicated SNP markers in most LGs were well ordered. The collinearity of each LG with the peanut reference genome was also analyzed. As shown in Figure S2, a relatively high collinearity was observed between 20 LGs and the reference genome.

### Phenotypic variation

**Figure 2:**
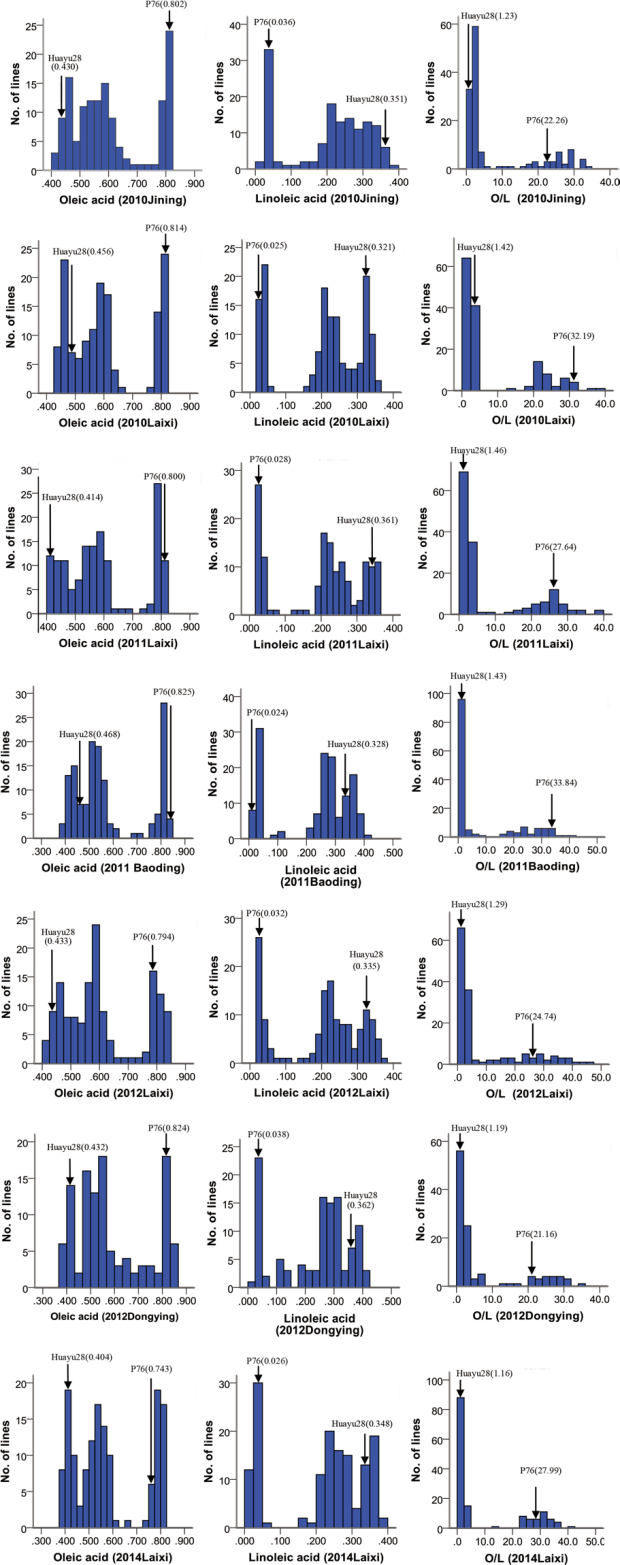
Phenotypic distribution of oleic acid, linoleic acid and O/L in RIL. The x-axis shows the ranges of oleic acid, linoleic acid and O/L over four years (2010, 2011, 2012, 2014) and the y-axis represents the number of individuals in the RIL population.

Phenotypic data for four seasons were generated for oleic acid, linoleic acid and O/L (the ratio of oleic acid (C18:1) to linoleic acid (C18:2)) on two parents and the complete set of RIL. Phenotypic analysis revealed that the fitted curves were found to be multimodal (oleic acid, linoleic acid and O/L). The oleic acid content (C18:1), linoleic acid content (C18:2) and the ratio of oleic acid (C18:1) to linoleic acid (C18:2) (O/L) of maternal parent, Huayu28, varied between 0.404~0.468, 0.321~0.362 and 1.146~1.426. The RIL showed high phenotypic variation for C18:1, C18:2 and O/L in seven environments. The C18:1 of RIL ranged between 0.401~0.819, 0.426~0.821, 0.404~0.818, 0.387~0.836, 0.405~0.843, 0.377~0.843, and 0.383~0.824 during 2010, 2011, 2012 and 2014. The C18:2 of RIL ranged between 0.024~0.390, 0.021~0.364, 0.021~0.362, 0.020~0.400, 0.018~0.368, 0.025~0.413, and 0.019~0.384 during 2010, 2011, 2012 and 2014. The O/L of RIL ranged between 1.028~33.871, 1.170~38.371, 1.123~38.451, 0.967~40.360, 1.100~45.966, 0.927~34.147, and 1.061~40.803 during 2010, 2011, 2012 and 2014 (Fig. 2).

### QTL analysis

Multi-season phenotypic data together with genotypic data generated on RIL were used for conducting QTL analysis. An interval mapping model with a logarithm of odds (LOD) score of 2.5 for potential QTLs was used for QTL detection. In total, significant QTLs for C18:1, C18:2 and O/L were found to be distributed on A03, A04, A09, B03, B09 and B10 of the peanut map (Table 3). For oleic acid (C18:1), two main QTLs were identified, which were located in A09 and B09 and showed consistent high contributions in seven environments. Phenotypic variation explained (PVE) of QTL in A09 ranged from 7.91% to 13.12% in seven environments, while PVE of QTL in B09 ranged from 46.15% to 57.60% in seven environments. QTL in A03 was identified in two environments (2012 Laixi and 2014 Laixi), and QTL in B10 was identified in two environments (2010 Jining and 2011 Laixi). For linoleic acid (C18:2) and O/L, similar results were found in seven environments. For all QTL for oleic acid, linoleic acid and O/L, the ‘P76’ parent contributed to high oleic acid (C18:1) and high O/L, while the ‘Huayu28’ parent contributed to high linoleic acid (C18:2).

**Table 3:** QTL information of oleic acid, linoleic acid and O/L in peanut in seven environments.

Trait	Environment	LG	Pos(cM)	Left-Right Marker	LOD	PVE(%)	Add	CI
C18:1	E1(2010-Jining)	B09	118	Marker2575339- Marker2379598	24.85	53.99	−0.09	116.5–119.5
		A09	62	Marker4391589- Marker4463600	5.16	7.91	−0.04	61.5–62.5
		B10	42	Marker3051079- Marker3146388	2.62	3.78	−0.02	36.5–43.5
	E2(2010-Laixi)	B09	117	Marker2575339- Marker2379598	28.19	55.29	−0.09	116.5–119.5
		A09	62	Marker4391589- Marker4463600	7.58	10.44	−0.04	61.5–62.5
	E3(2011-Laixi)	B09	117	Marker2575339- Marker2379598	31.85	57.60	−0.10	116.5–119.5
		A09	62	Marker4391589- Marker4463600	8.73	10.68	−0.04	61.5–62.5
		B10	42	Marker3051079- Marker3146388	3.33	3.68	−0.02	35.5–43.5
	E4(2011-Baoding)	B09	118	Marker2575339- Marker2379598	23.67	51.78	−0.10	116.5–119.5
		A09	62	Marker4391589- Marker4463600	5.60	8.74	−0.04	61.5–62.5
	E5(2012-Laixi)	B09	118	Marker2575339- Marker2379598	32.64	57.44	−0.10	116.5–119.5
		A09	62	Marker4391589- Marker4463600	7.53	8.60	−0.04	61.5–62.5
		A03	124	IPAHM_103- Marker4016934	3.42	3.98	−0.03	118.5–128.5
		A04	0	Marker5056376- Marker5054465	2.93	3.05	−0.02	0–2.5
	E6(2012-Dongying)	B09	118	Marker2575339- Marker2379598	18.28	46.15	−0.10	116.5–119.5
		A09	62	Marker4391589- Marker4463600	6.92	13.12	−0.05	60.5–63.5
	E7(2014-Laixi)	B09	117	Marker2575339- Marker2379598	30.44	55.66	−0.10	116.5–118.5
		A09	62	Marker4391589- Marker4463600	8.41	10.46	−0.04	60.5–62.5
		A03	124	IPAHM_103- Marker4016934	3.63	4.63	−0.03	118.5–128.5
C18:2	E1(2010-Jining)	B09	118	Marker2575339- Marker2379598	24.68	53.93	0.08	116.5–119.5
		A09	62	Marker4391589- Marker4463600	4.79	7.36	0.03	61.5–62.5
		B10	42	Marker3051079- Marker3146388	2.71	3.94	0.02	36.5–43.5
	E2(2010-Laixi)	B09	117	Marker2575339- Marker2379598	27.40	54.87	0.08	116.5–119.5
		A09	62	Marker4391589- Marker4463600	6.84	9.61	0.03	61.5–62.5
	E3(2011-Laixi)	B09	117	Marker2575339- Marker2379598	31.47	57.56	0.08	116.5–119.5
		A09	62	Marker4391589- Marker4463600	8.08	10.00	0.03	61.5–62.5
		B10	42	Marker3051079- Marker3146388	3.23	3.64	0.02	35.5–43.5
	E4(2011-Baoding)	B09	118	Marker2575339- Marker2379598	23.46	51.87	0.09	116.5–119.5
		A09	62	Marker4391589- Marker4463600	5.05	7.92	0.04	61.5–62.5
	E5(2012-Laixi)	B09	117	Marker2575339- Marker2379598	29.61	56.78	0.08	116.5–119.5
		A09	62	Marker4391589- Marker4463600	7.20	9.35	0.03	61.5–62.5
		A03	124	IPAHM_103- Marker4016934	2.92	3.85	0.02	118.5–130
	E6(2012-Dongying)	B09	117	Marker2575339- Marker2379598	18.38	46.33	0.08	116.5–119.5
		A09	62	Marker4391589- Marker4463600	6.67	12.95	0.05	60.5–63.5
	E7(2014-Laixi)	B09	117	Marker2575339- Marker2379598	29.89	55.65	0.09	116.5–118.5
		A09	62	Marker4391589- Marker4463600	7.13	8.96	0.04	60.5–62.5
		A03	124	IPAHM_103- Marker4016934	3.94	5.16	0.03	116.5–128.5
O/L	E1(2010-Jining)	B09	118	Marker2575339- Marker2379598	13.84	36.50	−6.57	116.5–120.5
	E2(2010-Laixi)	A09	62	Marker4391589- Marker4463600	3.38	6.71	−2.61	60.5–62.5
		B09	118	Marker2575339- Marker2379598	15.64	39.65	−6.35	116.5–119.5
	E3(2011-Laixi)	A09	62	Marker4391589- Marker4463600	3.84	7.34	−2.85	60.5–62.5
		B09	118	Marker2575339- Marker2379598	16.80	41.07	−6.74	116.5–120.5
	E4(2011-Baoding)	A09	62	Marker4391589- Marker4463600	2.88	5.86	−2.79	60.5–63.5
		B09	118	Marker2575339- Marker2379598	15.20	39.51	−7.24	116.5–120.5
	E5(2012-Laixi)	A04	114	Marker4982682- Marker4932636	2.57	5.28	−2.65	113.5–114.5
		A09	62	Marker4391589- Marker4463600	2.87	6.00	−2.74	60.5–62.5
		B09	117	Marker2575339- Marker2379598	14.82	38.33	−6.92	116.5–119.5
	E6(2012-Dongying)	B03	83	Marker4742410- Marker4872231	4.23	10.10	−3.15	82.5–83.5
		A09	62	Marker4391589- Marker4463600	4.59	11.13	−3.11	60.5–63.5
		B09	117	Marker2575339- Marker2379598	8.88	23.87	−4.53	116.5–119.5
	E7(2014-Laixi)	A09	62	Marker4391589- Marker4463600	3.59	6.28	−3.17	60.5–63.5
		B09	118	Marker2575339- Marker2379598	18.68	43.41	−8.34	116.5–119.5

QTL: quantitative trait loci; LG: linkage group; LOD: likelihood of odds; PVE: phenotypic variance explained of each QTL; Add: additive value; CI: confidence interval.

## Discussion

The SLAF-seq strategy, a combination of locus-specific amplification and high-throughput sequencing, has been subjected to a series of critical trials to guarantee its high efficiency, accuracy and density^20^. This approach has been successfully applied to high-density genetic map construction, QTL analysis and mapping genes for many plants and animals^23–35^. Based on a careful analysis of the genomic GC content, repeat conditions and genome length of *Arachis duranensis* and *Arachis ipaensis*, HaeIII was selected to digest the genomic DNA with a digestion rate of 94.02%. Subsequently, SLAFs (314–394 bp) were selected in a pilot experiment for further paired-end sequencing. A pre-designed scheme and a pilot experiment were conducted to ensure the density, uniformity and efficiency of the marker development. The average genotype quality score of all SLAF markers reached the cut-off value of 30, which was sufficient to filter the reads with low sequencing depth. Thus, the combination of sequence depth and genotype quality scores sufficiently enhanced the genotyping accuracy. Using high-throughput SLAF sequencing, we developed 433,679 high-quality SLAFs, of which 29,075 were polymorphic. A total of 2,266 polymorphic SLAFs were identified for linkage map construction. The sequencing average depth of these SLAFs in the parents and progenies were 54.32-fold, 57.72-fold and 9.83-fold, respectively. Our results clearly demonstrate that SLAF-seq is efficient for large-scale genotyping and rapid development of a large number of genetic markers. DNA marker distribution is not random with some genomic regions highly populated with markers whereas others are under-represented. In the present map, inter-marker distances varied in sizes. The inter-marker distances on A01, A02, A04, A05, A07, A09, B03, B04, B05 and B08 were more than 90%, which were less than and/or equal to 5.0 cM. The inter-marker distances less than and/or equal to 5.0 cM on other chromosomes were less than 90%, and these chromosomes comprised of less than 74 markers. Inter-marker distances larger than 10.0 cM were found on all chromosomes except A05, A09 and B05, suggesting that such inter-marker distances are not restricted to a particular chromosome. The longest one was 26.24 cM on the distal end of B02. The presence of these inter-marker distances may have negative effects on the application of mapped DNA markers; for example, genomic regions that lack DNA markers will make detection of quantitative trait loci (QTL) difficult^36^. Therefore, more comparable markers between different peanut maps are needed to fill in the inter-marker distances to obtain a more complete coverage of the peanut genome. A comparison between the high-density genetic linkage map and the maps from Huang *et al*. and Peanutbase showed that 15 SSR markers were mapped to the same linkage group, 9 SSR markers showed different results to those of previous maps and 12 SSR markers showed confused positions. For example, TC13E05 was mapped in A02 in this study, while it was located in B02 of Peanutbase. IPAHM_93 was mapped in A03 of both the maps in this study and Huang *et al*., but was mapped in B03 of Peanutbase. Ah1TC6H03, IPAHM_659 and Ah1TC5D06 showed similar results. The AA and BB subgenomes are highly similar^19^ which may contribute to the difficulty of marker assignment to the subgenomes.

Segregation distortion is a common biological phenomenon and is one of the engines driving evolutionary processes. It can be observed in almost all types of hybrid segregating populations. In general, the skewed segregation ratio of RIL populations is higher than that of backcross populations (BC) and doubled haploid populations (DH). F_2_ populations show the lowest skewed segregation ratio^37^. The genetic basis of segregation distortion is still under debate, and gametophyte and/or zygotic selection and chromosomal rearrangements may be the main causes^38^. Studies have demonstrated a large number of segregation distortions in many species, such as maize (*Zea mays*)^39^, barley (*Hordeum vulgare*)^40^, potato (*Solanum tuberosum*)^41^, sesame^23^, and sorghum (*Sorghum bicolor*)^38,42,43^. Segregation distorted markers are common in peanut linkage maps. Hong *et al*. (2010) used three RIL populations to construct a composite linkage maps with 175 SSR markers. The total composite map length is 885.4 cM, with an average marker density of 5.8 cM. Segregation distortion in the three populations was 23.0%, 13.5% and 7.8% of the markers, respectively^4^. Zhou *et al*. (2014) constructed one linkage map which was comprised of 1,685 marker loci, including 1,621 SNPs and 64 simple sequence repeat (SSR) markers, of which 659 markers showed significant segregation distortion and were distributed among every LG^10^. Huang *et al*. (2016) used a RIL population to construct a high-density genetic linkage map with 1,219 mapped loci covering a total map length of 2,038.75 cM. Chi-square (χ^2^) analysis revealed significant segregation distortion *(p* < 0.05) for 152 loci (12.5%)^15^. In the present study, a RIL mapping population was used to construct a linkage map, and 700 markers (29.99%) of the 2,334 assigned markers showed significant segregation distortion. Most skewed loci were located on A04, B03, and B05. Although the molecular mechanism of segregation distortion remain undiscovered, numerous studies have shown that segregation distortion markers for linkage map construction could increase the quantity of markers on the map, the genome coverage of the map, and help to improve the detection of linked QTLs^23,44–46^. Xu showed that distorted markers can be used for QTL mapping with no detrimental effect on the result and can be beneficial if used properly^44^. Zhang *et al*. showed that segregation distortion could result in higher genetic variance than non-distortion and help to improve the detection of linked QTLs because distortion markers do not have a large effect on the position or effect estimations of QTL analysis^46^. Zhang *et al*. (2013) mapped 205 markers of segregation distortion onto the final map with a distribution on every LG of sesame, similar to the distribution of all markers^23^.

Oleic acid (C18:1) and linoleic acid (C18:2) were the major fatty acids and accounted for about 80% of peanut oil. Oleic acid (C18:1) is about 47% in normal and up to 80% in high oleic peanut lines and is associated with several human health benefits^47,48^. Linoleic acid (C18:2) is about 40% in normal and down to 2% in high oleic peanut lines. Manish *et al*. (2014) used RIL populations derived from normal and high oleic peanut varieties to locate *FAD2* genes for C18:1, C18:2 and oleic/linoleic acid ratio (O/L)^11^. The mapping positions for *ahFAD2A* (A sub-genome) and *ahFAD2B* (B sub-genome) genes were assigned on A09 and B09 linkage groups. The PVE of *ahFAD2B* for C18:1, C18:2 and O/L were 26.54%, 25.59% and 41.02%, and the PVE of *ahFAD2A* for C18:1, C18:2 and O/L were 8.08%, 6.86% and 3.78%. In this study, we detected the two main QTLs for C18:1, C18:2 and O/L being located in A09 and B09. PVE of QTLs for C18:1, C18:2 and O/L in B09 ranged from 46.15% to 57.60%, 46.33% to 57.56% and 23.87% to 43.41%, respectively, while in A09 ranged from 7.19% to 13.12%, 7.36% to 12.95% and 6% to 11.13%, respectively. Genomic approaches such as high-throughput sequencing and large-scale genotyping technologies have been used in genetic linkage mapping. The SLAF-seq method provided significant advantages to generate enough polymorphic markers for high-density genetic map construction in peanut. The high density map is sufficient to ensure adequate polymorphic marker coverage in regions of interest and can be used as a reference map for peanut genetic studies.

## Conclusions

In this study, we used the SLAF-seq method to develop 433,679 high-quality SLAFs, of which 29,075 were polymorphic. To our knowledge, we have constructed the first high-density genetic map of SLAF for the cultivated peanut (*A. hypogaea* L.), which consisted of 2,334 markers (68 SSRs and 2,266 SNPs) on the 20 linkage groups spanning 2,586.37 cM. The average distance between adjacent markers was 2.25 cM. The analysis of the SLAFs and their sequence information identified that SLAF-seq is an effective strategy for large-scale genotyping applied to the construction of a high-density map of peanut. Based on phenotyping in seven environments, QTLs for oleic acid (C18:1), linoleic acid (C18:2) and the ratio of oleic acid to linoleic acid (O/L) were identified and positioned on linkage groups A03, A04, A09, B09 and B10. Marker2575339 and Marker2379598 in B09 were associated with C18:1, C18:2 and O/L in seven environments, and Marker4391589 and Marker4463600 in A09 were associated with C18:1, C18:2 and O/L in six environments. This map exhibits high resolution and accuracy. It will facilitate QTL discovery for essential agronomic traits in peanut.

## Material and Methods

### Plant materials and DNA extraction

An F_2:11_ population of 146 RILs was derived from a cross between ‘Huayu28’ and ‘P76’. ‘Huayu28’ was early-mature, normal oleic content and small-seed peanut variety. ‘P76’ was lately-mature, high oleic content and medium-seed peanut variety. Seedlings of progeny and parents were planted in the experiment field of Shandong Peanut Research Institute in Laixi, Shandong Province, China, in 2014. Young healthy leaves from two parents and 146 RILs (F_2:11_) were collected and frozen in liquid nitrogen, then transferred to a −70 °C freezer. Total genomic DNA was extracted from each leaf sample by Plant Genomic DNA Kit (Tiangen Biotech (Beijing) Co., Ltd.). The concentration and quality of DNA were examined by electrophoresis in 0.8% agarose gels with a standard lambda DNA, and an ND-1000 spectrophotometer (NanoDrop, Wilmington, DE, USA).

### SLAF library construction and high-throughput sequencing

SLAF-seq was used to genotype the 146 RILs and the two parents, as previously described^13^, with a few modifications. In brief, genomic DNA from each sample was treated with HaeIII (NEB, Ipswich, MA, USA), T4 DNA ligase (NEB), ATP (NEB), and HindIII adapter at 37 °C. These restriction-ligation reaction solutions were diluted and mixed with dNTP, Taq DNA polymerase (NEB) and MseI primer containing barcode 1 for PCR reactions. The E.Z.N.A Cycle Pure Kit (Omega, London, UK) was used to purify the PCR products. The purified PCR products were pooled and incubated at 37 °C with MseI, T4 DNA ligase, ATP, and Solexa adapter. After incubation, the reaction products were then purified using a Quick Spin column (Qiagen, Venlo, Netherlands), and electrophoresed on a 2% agarose gel. SLAFs of 314–394 bp (including adapter sequence indexes and adaptors) in size were selected for paired-end sequencing on a Gel Extraction Kit (Qiagen). The gel-purified product was sequenced on the Illumina HiSeq. 2500 system (Illumina, Inc; San Diego, CA, U.S.) according to the manufacturer’s recommendations. Real-time monitoring was performed for each cycle during sequencing, and the ratio of raw high-quality reads with quality scores greater than Q30 (a quality score of 30 indicates a 0.1% chance of obtaining an error, and thus 99.9% confidence) and the guanine-cytosine (GC) content were calculated for quality control. All sequences clustered together were defined as a SLAF loci. In each of the SLAF, we found polymorphism loci between the parents, and most of these were SNPs. All polymorphism SLAFs loci were genotyped with consistency in the offspring and parental SNP loci.

### SLAF and SSR data analysis and genotyping

SLAF-seq data was operated using the software developed by Sun *et al*.^20^, and the genotyping methods with reference to Sun *et al*. (2013) and Wei *et al*.^27^. According to sequence similarity, the generated pair-end reads from SLAF-seq were clustered, and the reads could be inferred from one-to-one alignment by BLAT (-tileSize = 10-stepSize = 5). Identical reads were merged, and the reads with over 90% similarity sequences were grouped into one SLAF locus^20^. In each SLAF locus, minor allele frequency (MAF) evaluation was used to define alleles.

In order to ensure the quality of the genetic map, the following rules were applied to filter SLAFs: (1) removal of SLAFs with parents sequence depth of less than 10×; (2) removal of SLAFs with complete degree below 70%; (3) removal of SLAFs with serious distorted segregation *(p-*value < 0.01); (4) removal of SLAFs with more than eight SNPs. SLAFs that passed the four-step filtering process were considered as potential markers. Those polymorphic SLAF markers were then assorted into eight segregation patterns as follows: ab×cd, ef×eg, hk×hk, lm×ll, nn×np, aa×bb, ab×cc, and cc×ab (Table 4). Since the RIL mapping populations were derived from two homozygous peanut varieties with a genotype of aa or bb, only the SLAF markers which had segregation patterns of aa×bb were used in map construction.

**Table 4:** Genotype of parents and offspring.

Type	Paternal genotype	Maternal genotype	Offspring genotype
ab × cd	ab	Cd	ac, ad, bc, bd,—
ef × eg	ef	Eg	ee, ef, eg, fg,—
ab × cc	ab	Cc	ac, bc,—
cc × ab	cc	Ab	ac, bc,—
hk × hk	hk	Hk	hh, hk, kk,—
lm × ll	lm	Ll	lm, ll,—
nn × np	nn	np	nn, np,—
aa × bb	aa	bb	aa, bb, ab,—

Note:–missing genotype of offspring.

SSR primers (Supplementary Supplementary Dataset 3) were selected from several previous articles. The PCR reactions conditions used were as follows: 3 min denaturation at 94 °C; 35 cycles of 1 min at 94 °C, 30 s at 55 °C, and 90 s at 72 °C; and then a final extension of 10 min at 72 °C and storage at 4 °C. The PCR products were separated on 8% PAGE gel. The segregation data for SSR markers in the same population were detected, and 68 of them were used for construction of the genetic map.

### High-density genetic map construction

Since next-generation sequencing data may include many genotyping errors and deletions, which could reduce the quality of the high-density linkage maps, High Map Strategy was used to order the SLAF and SSR markers, and to correct the genotyping errors in the linkage groups^49^. After genotyping of the 146 RILs, 2-point linkage analysis was performed for efficient markers. All high-quality SLAF and SSR markers were allocated to 20 LGs on the basis of their locations on chromosomes. A detailed MST map algorithm was used to order the SLAF and SSR markers^50^, and the SMOOTH algorithm was used to correct the genotyping errors as per the marker ordering^51^. All LGs were processed as follows: primary markers was used to order the LGs by their location on chromosomes; according to the relationship between the ordered markers, genotyping errors or deletions were corrected using the SMOOTH algorithm; the minimum spanning tree map was used to order the map; and the SMOOTH algorithm was used to correct the newly ordered genotypes. After four or more cycles of this processing, 20 high-quality maps were obtained. The Kosambi mapping function was used to estimate the map distances^52^.

### QTL analysis

Based on the integrated map, significant loci associated with oleic acid, linoleic acid and the ratio of oleic acid to linoleic acid were identified based on LOD scores larger than the 5% cutoff value determined through 1,000 permutation tests using the CIM method from the ‘qtl’ package of R.MapQTL6.0^53^, which was used to conduct logarithm of odds and percentage of phenotypic variance explained analysis, and interval mapping (IM)^54^ was used to detect QTLs for target traits. According to this method^55^, the fatty acids content was detected by gas chromatography (GC). Oleic and linoleic acids were investigated in 2010 (Laixi and Jining), 2011(Laixi and Baoding), 2012 (Laixi and Dongying) and 2014 (Laixi).

## Electronic supplementary material

Supplementary Information

Supplementary Dataset 1

Supplementary Dataset 1

Supplementary Dataset 2
