# Supplementary material for: High-Density Genetic Map Construction and Identification of QTLs Controlling Oleic and Linoleic Acid in Peanut using SLAF-seq and SSRs
Source: Sci Rep. 2018 Apr 3;8:5479. doi: 10.1038/s41598-018-23873-7 (PMC5883025; doi:10.1038/s41598-018-23873-7)
Supplement: Supplementary file 1 — Supplementary Information [file 41598_2018_23873_MOESM1_ESM.pdf]

High-Density Genetic Map Construction and Identification of QTLs Controlling Oleic and Linoleic Acid in Peanut using SLAF-seq and SSRs

X.H.Hu1*, S.Z.Zhang1*,H.R.Miao1*, F.G.Cui1, Y.Shen2, W.Q.Yang1, T.T.Xu1, N.Chen1, X.Y.Chi1, Z.M.Zhang, J.Chen1†,
1 Shandong Peanut Research Institute, Qingdao, 266100 P.R. China;
2 Institute of Industrial Crops, Jiangsu Academy of Agricultural Sciences,Nanjing, 210014, P.R. China
* Equal contributors.
†Corresponding author:mianbaohua2008@126.com

Figure S1

Peanut high density genetic map. The map was constructed based SLAF markers and SSR markers.

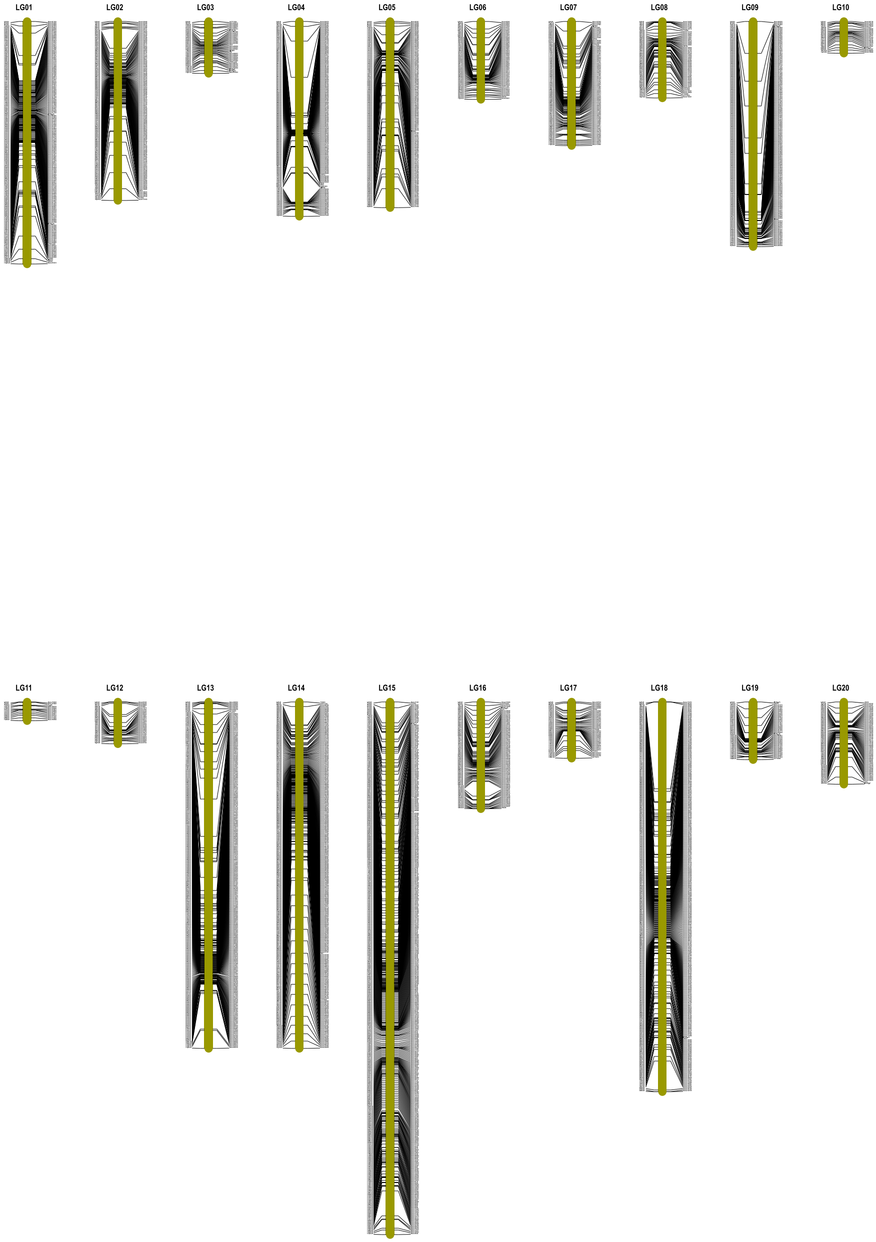
LG01
LG02
LG03
LG04
LG05
LG06
LG07
LG08
LG09
LG10
LG11
LG12
LG13
LG14
LG15
LG16
LG17
LG18
LG19
LG20

Figure S2: The correlation of the genetic and physical positions.

Axis of abscissa represents the genetic linkage group, axis of ordinate represents the physical positions.

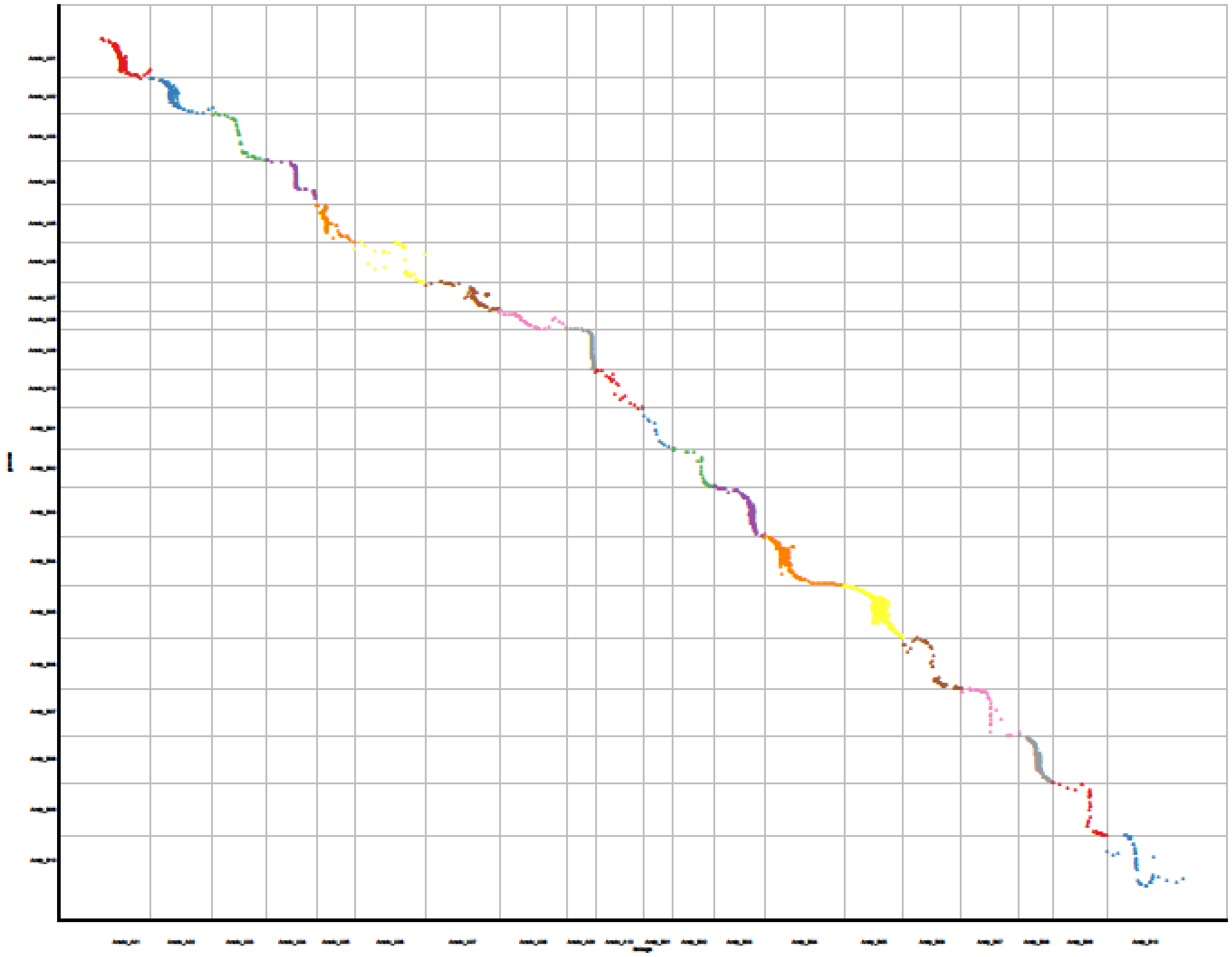

Addition file S1: Haplotype map of the genetic map

Haplotype map of the genetic map. Blue represents Huayu28, green represents P76, gray represents deletions, and red indicates heterozygosity.

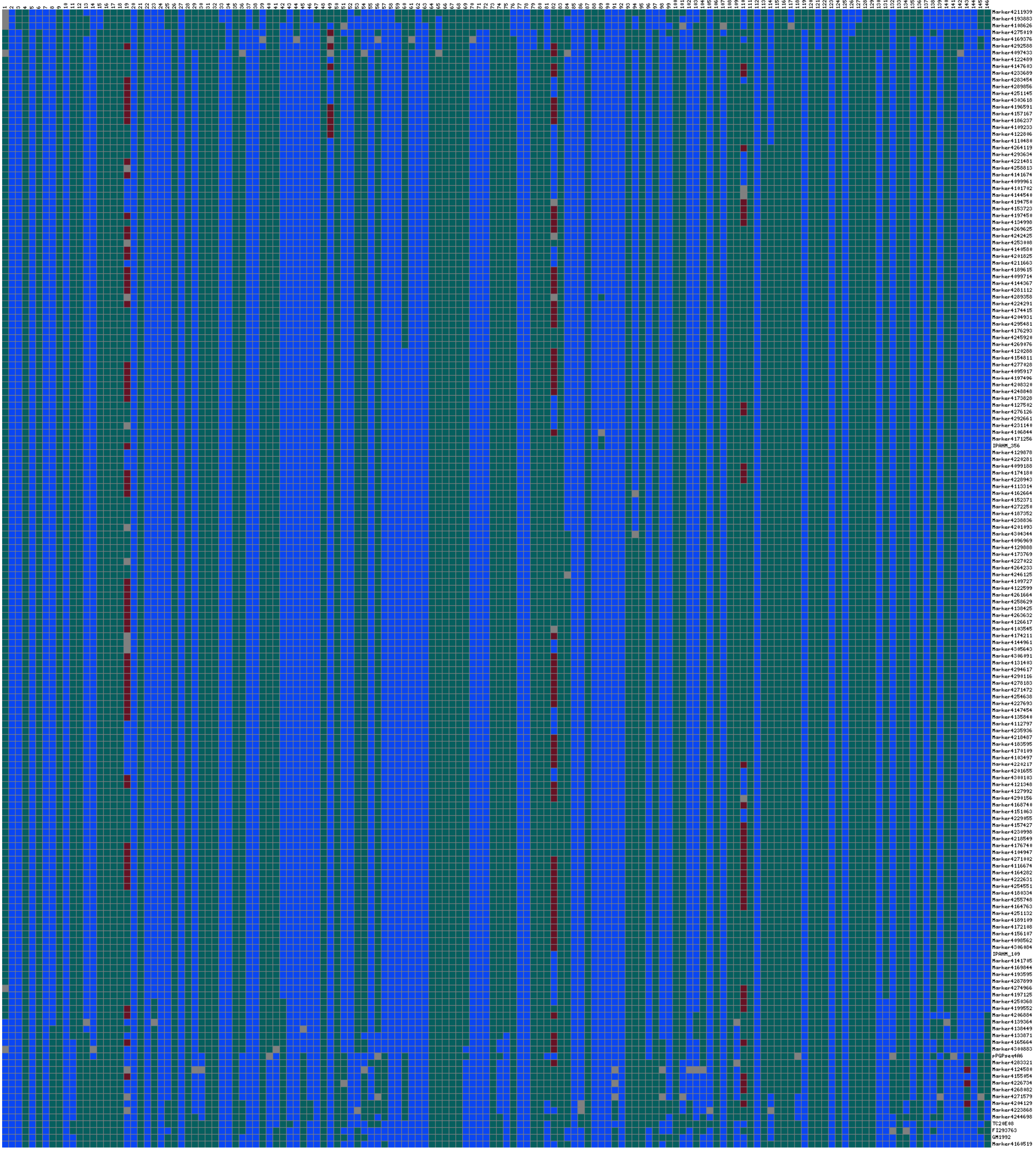

Haplotype map of the integrated map (Aradu_A01)

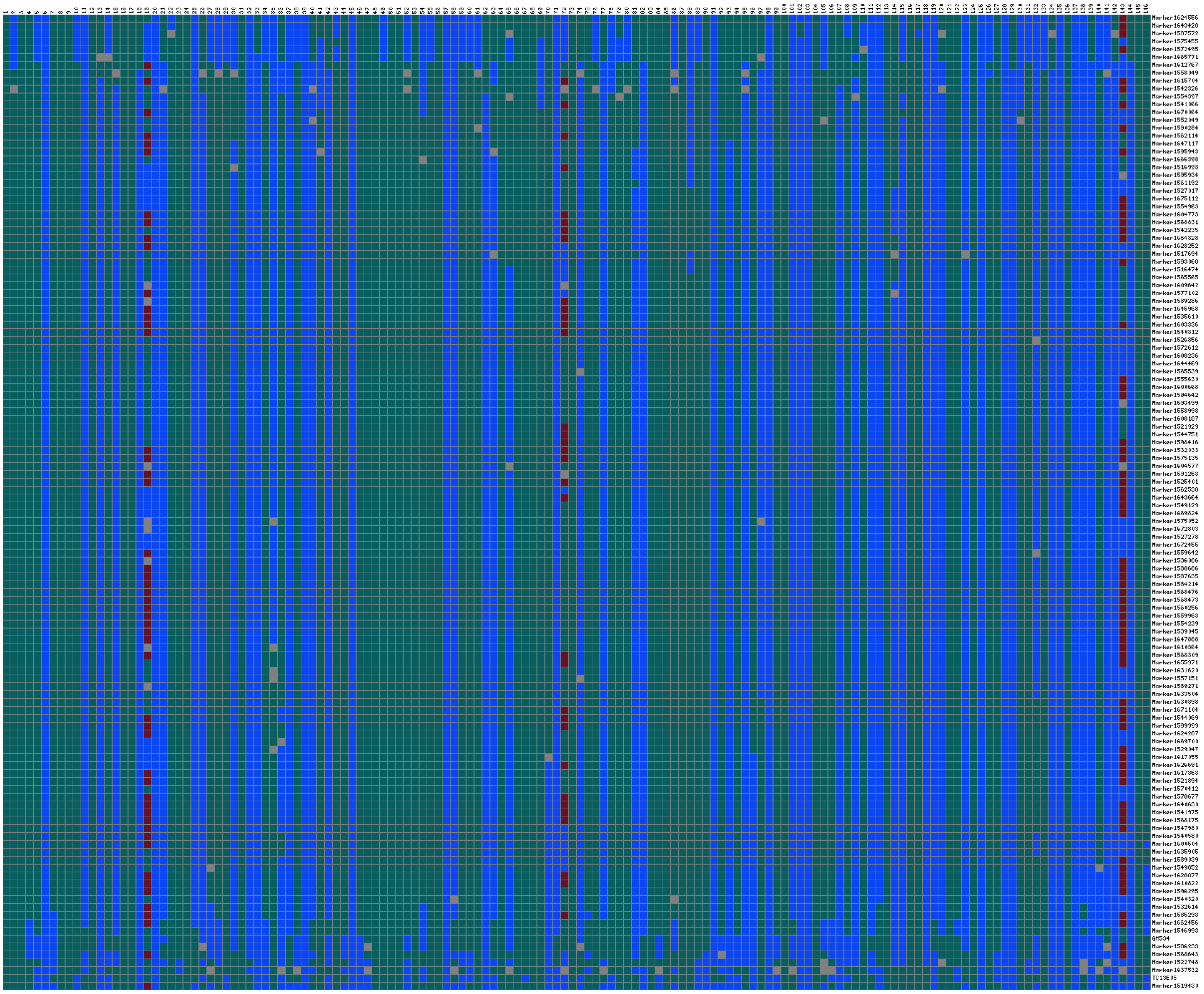

Haplotype map of the integrated map (Aradu_A02)

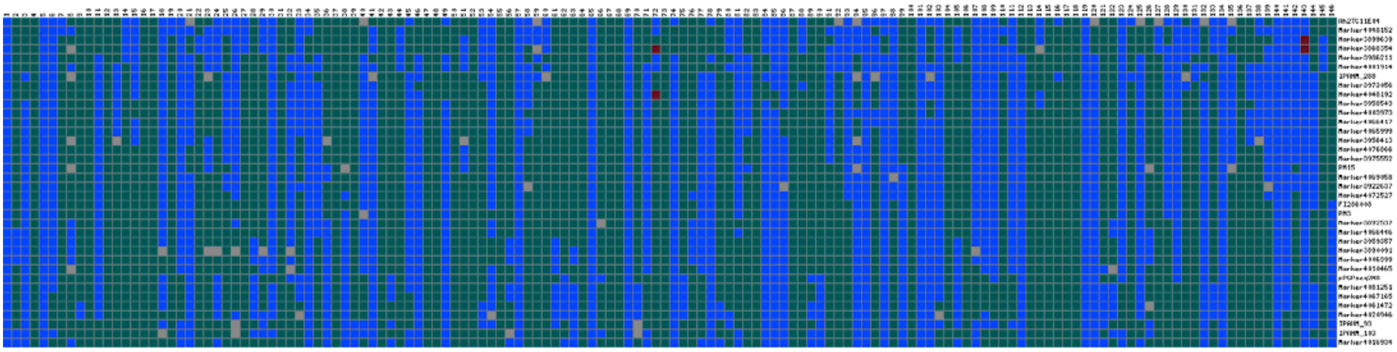

Haplotype map of the integrated map (Aradu_A03)

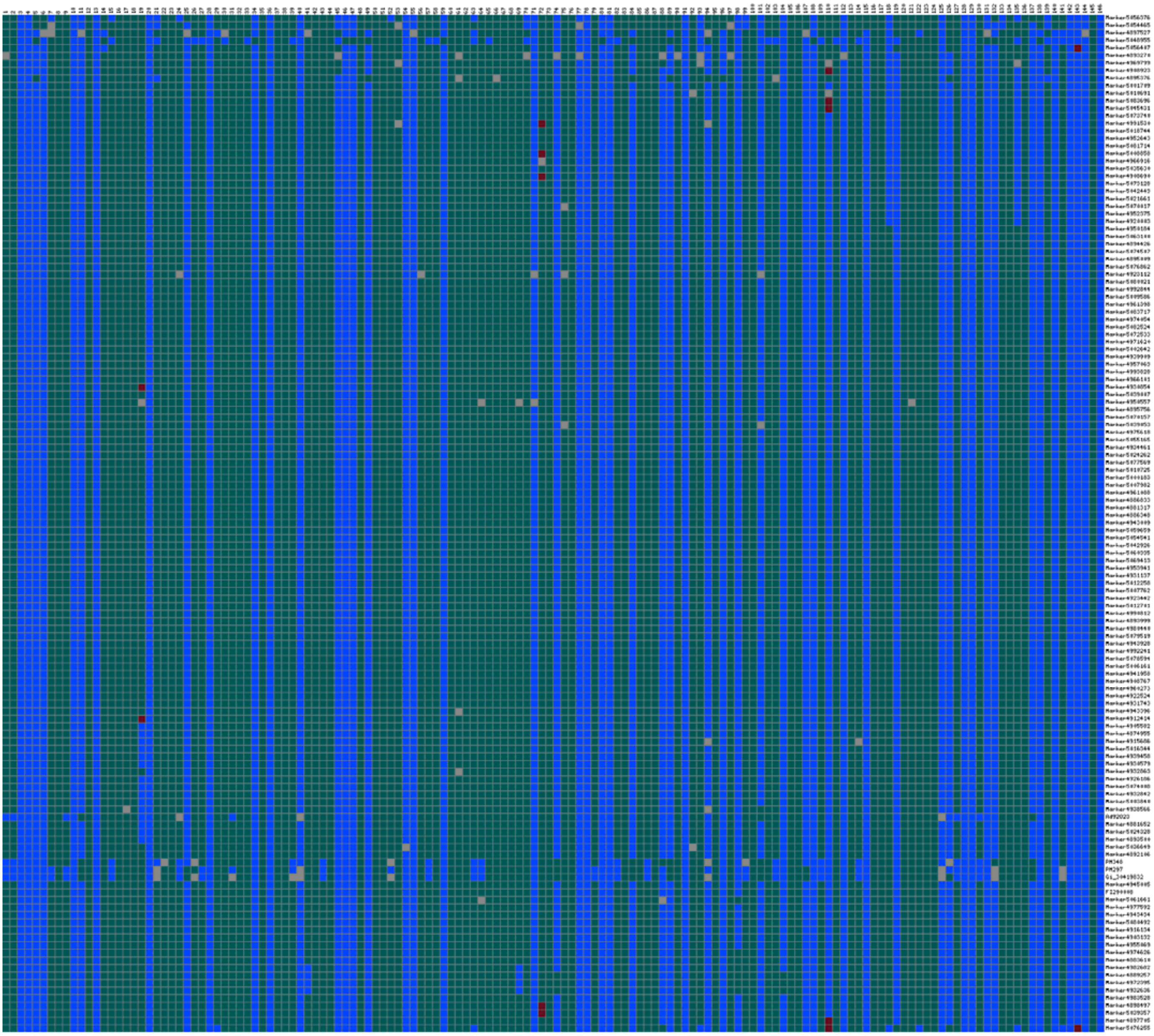

Haplotype map of the integrated map (Aradu_A04)

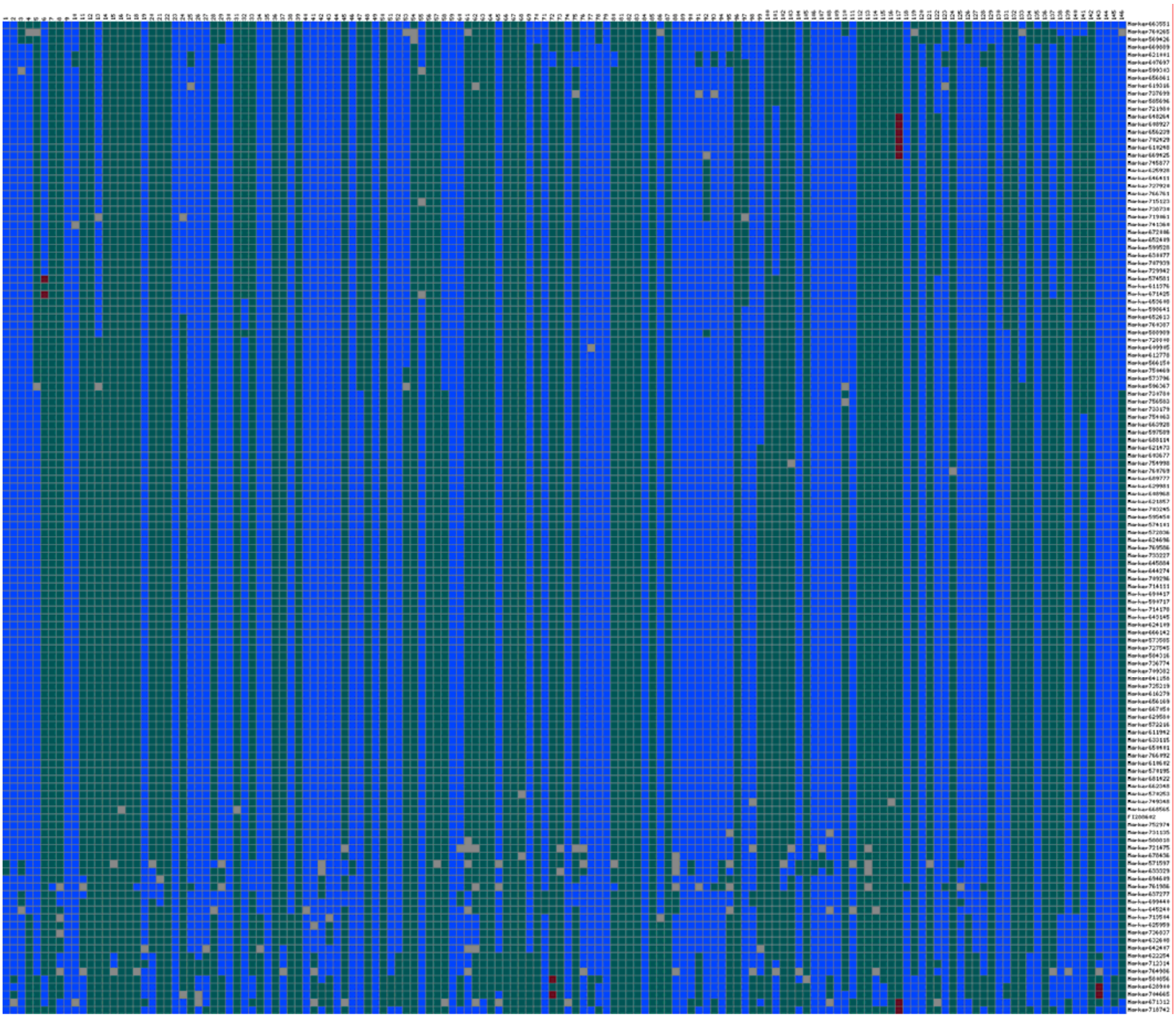

Haplotype map of the integrated map (Aradu_A05)

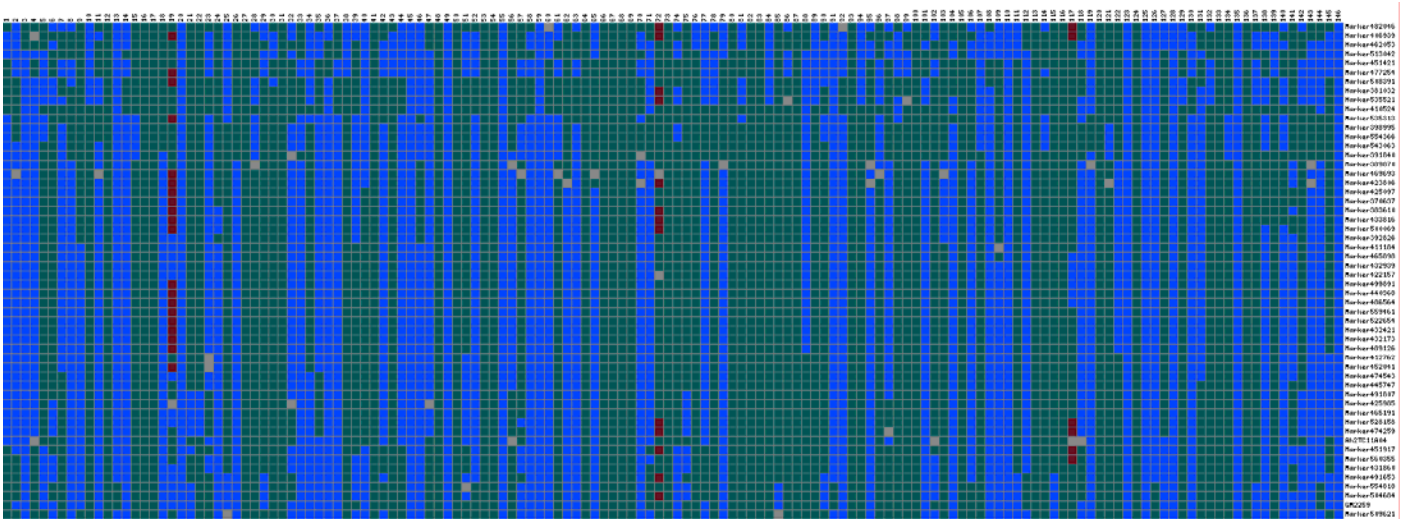

Haplotype map of the integrated map (Aradu_A06)

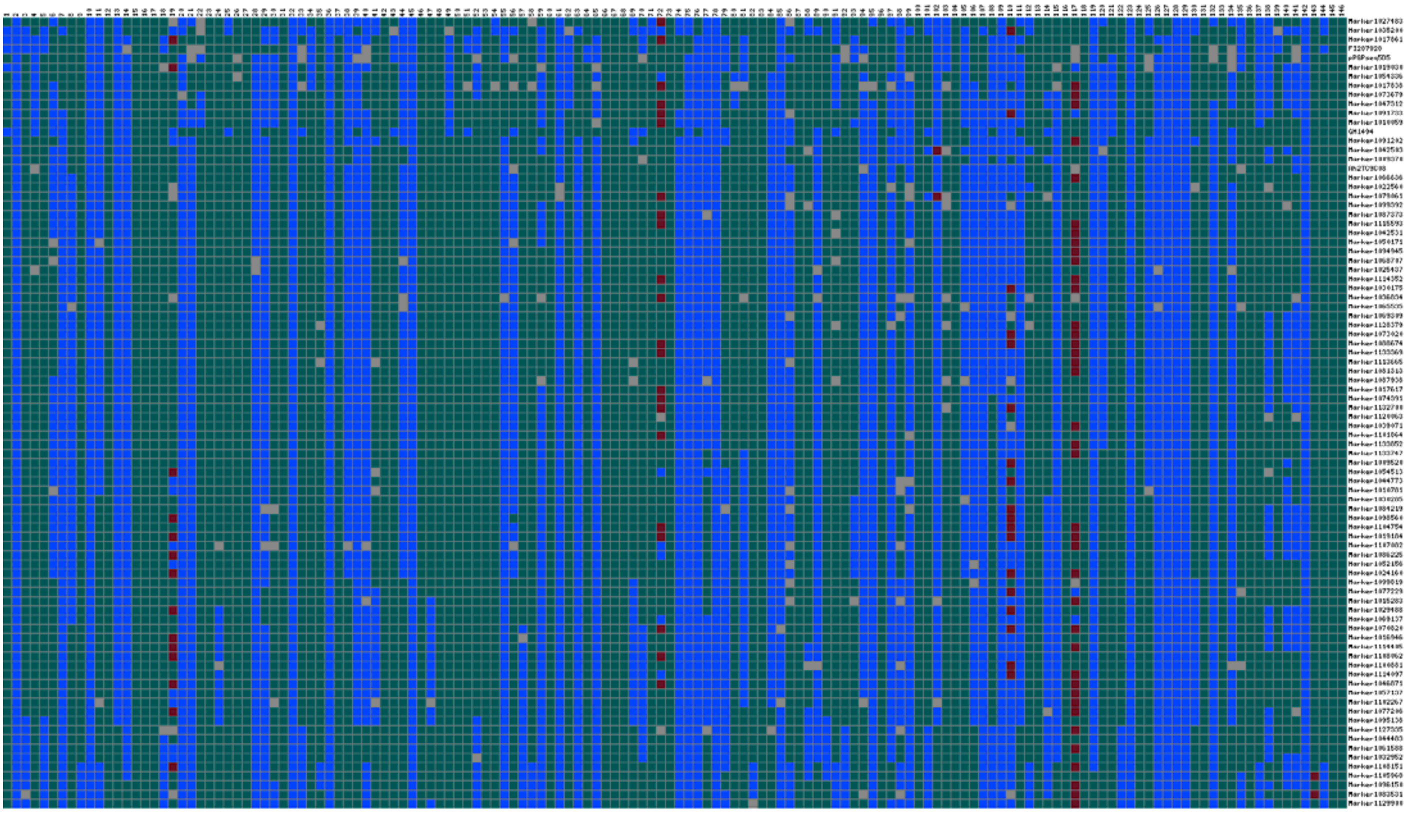

Haplotype map of the integrated map (Aradu_A07)

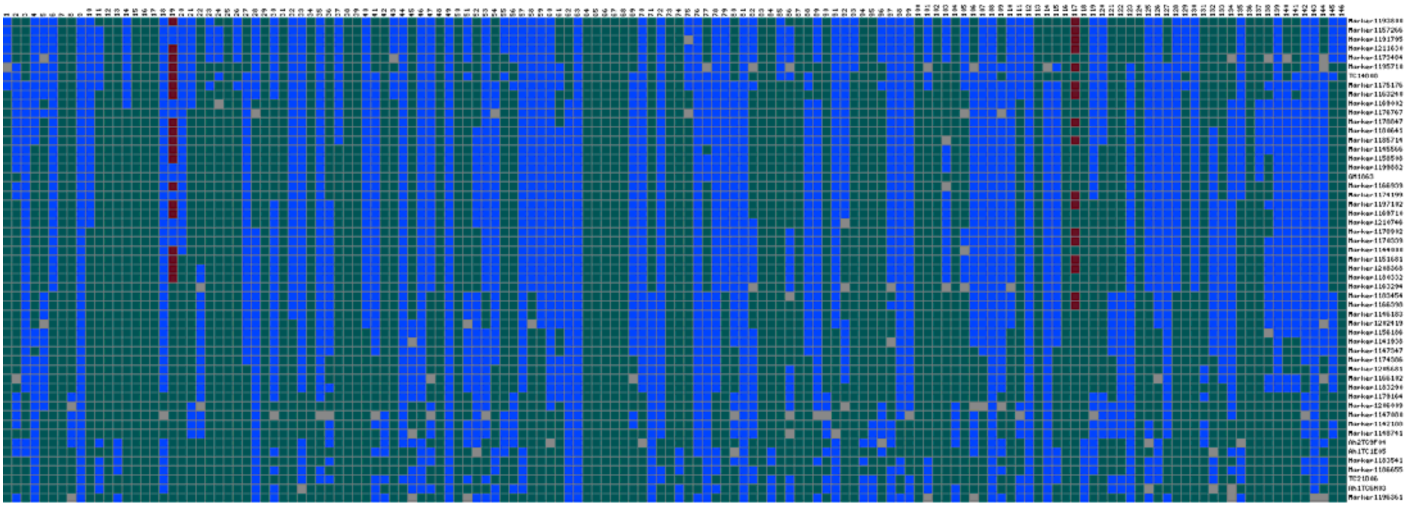

Haplotype map of the integrated map (Aradu_A08)

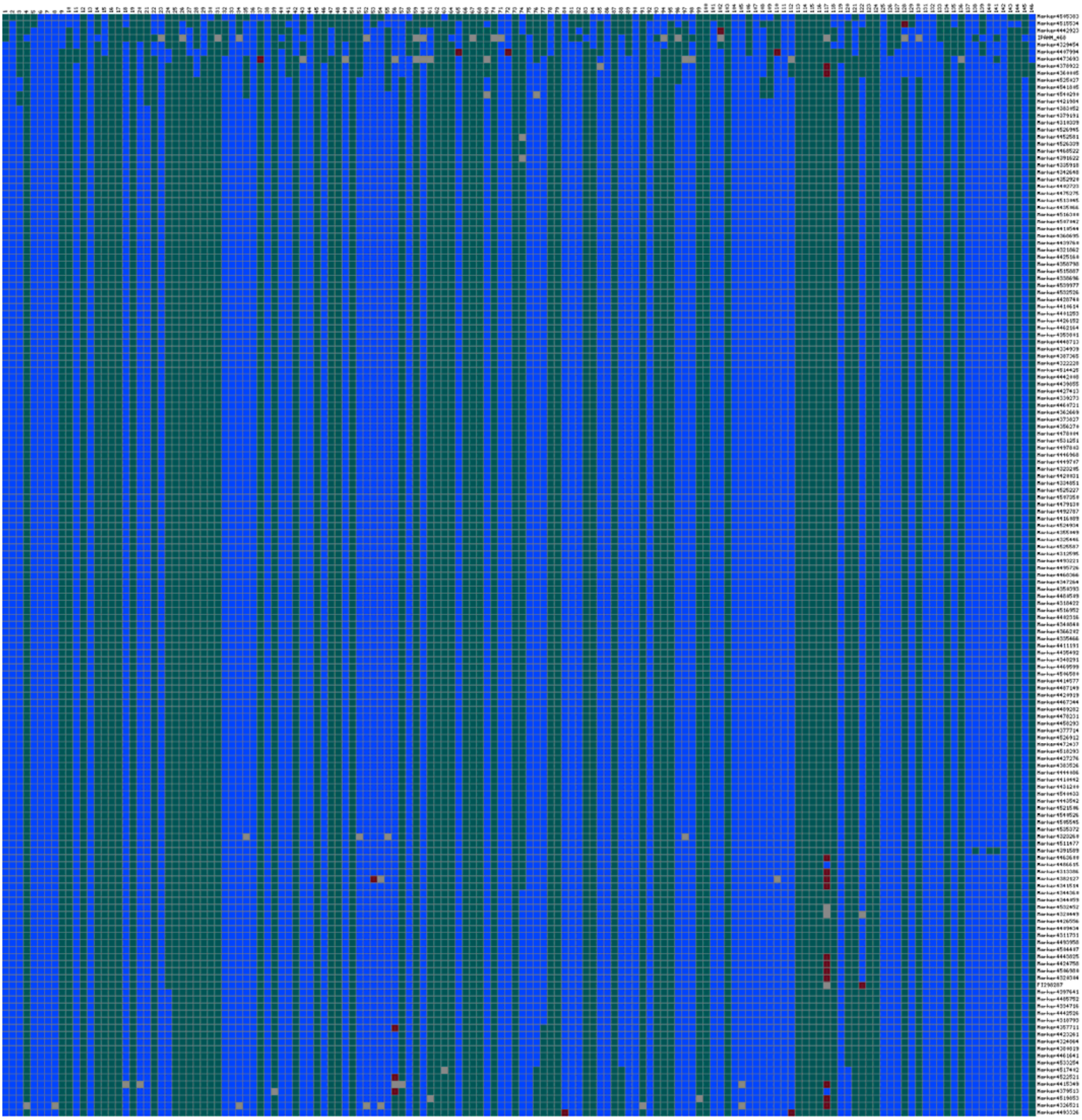

Haplotype map of the integrated map (Aradu_A09)

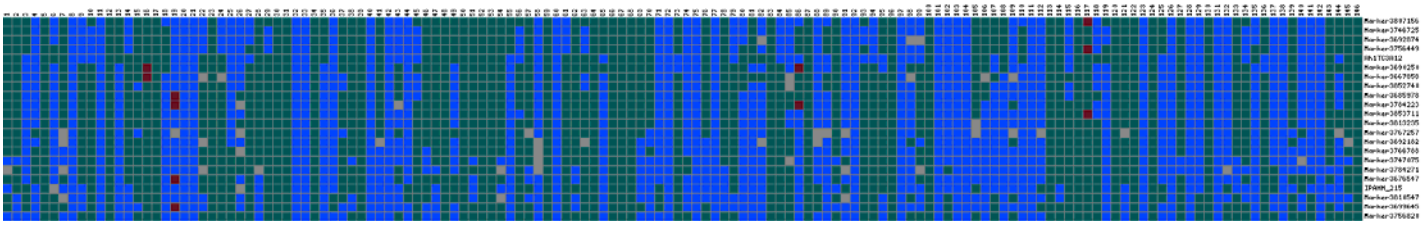

Haplotype map of the integrated map (Aradu_A10)

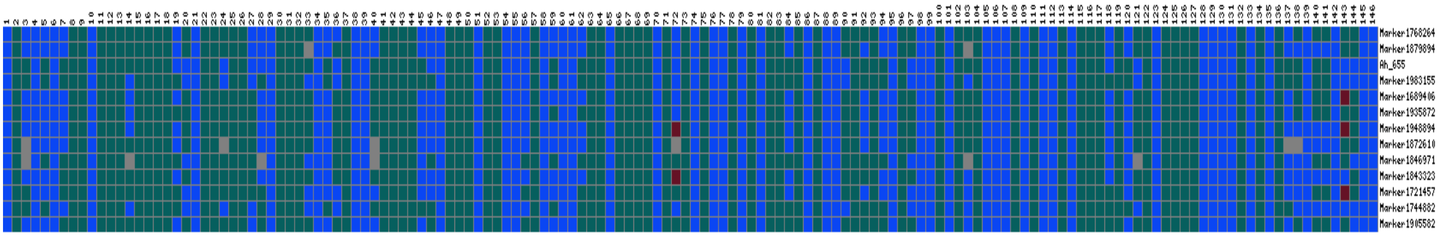

Haplotype map of the integrated map (Araip_B01)

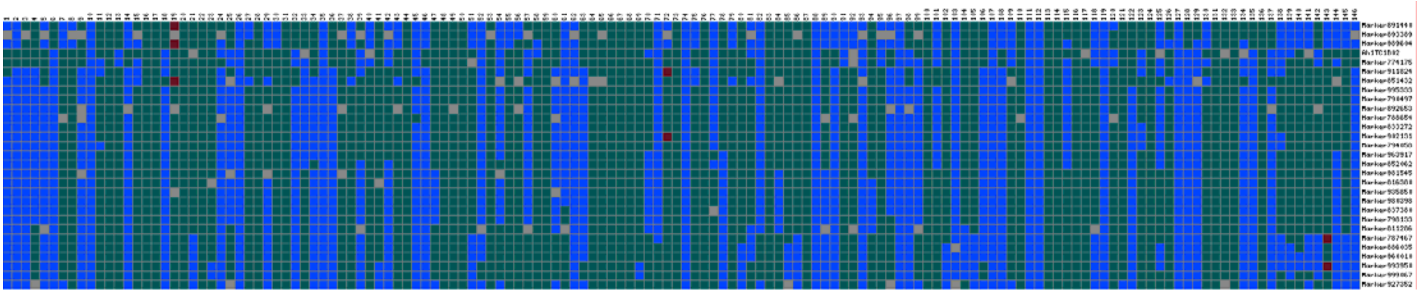

Haplotype map of the integrated map (Araip_B02)

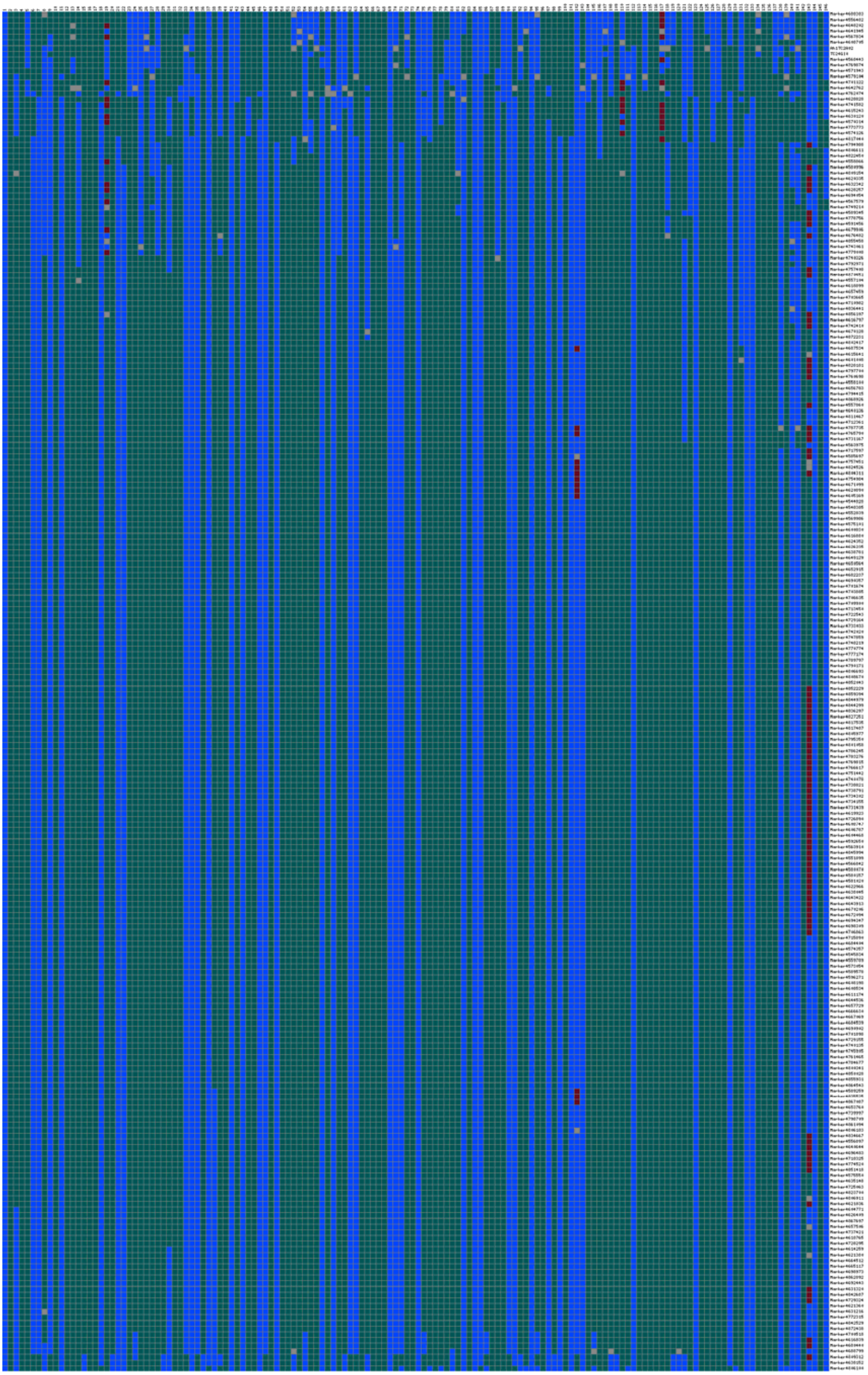

Haplotype map of the integrated map (Araip_B03)

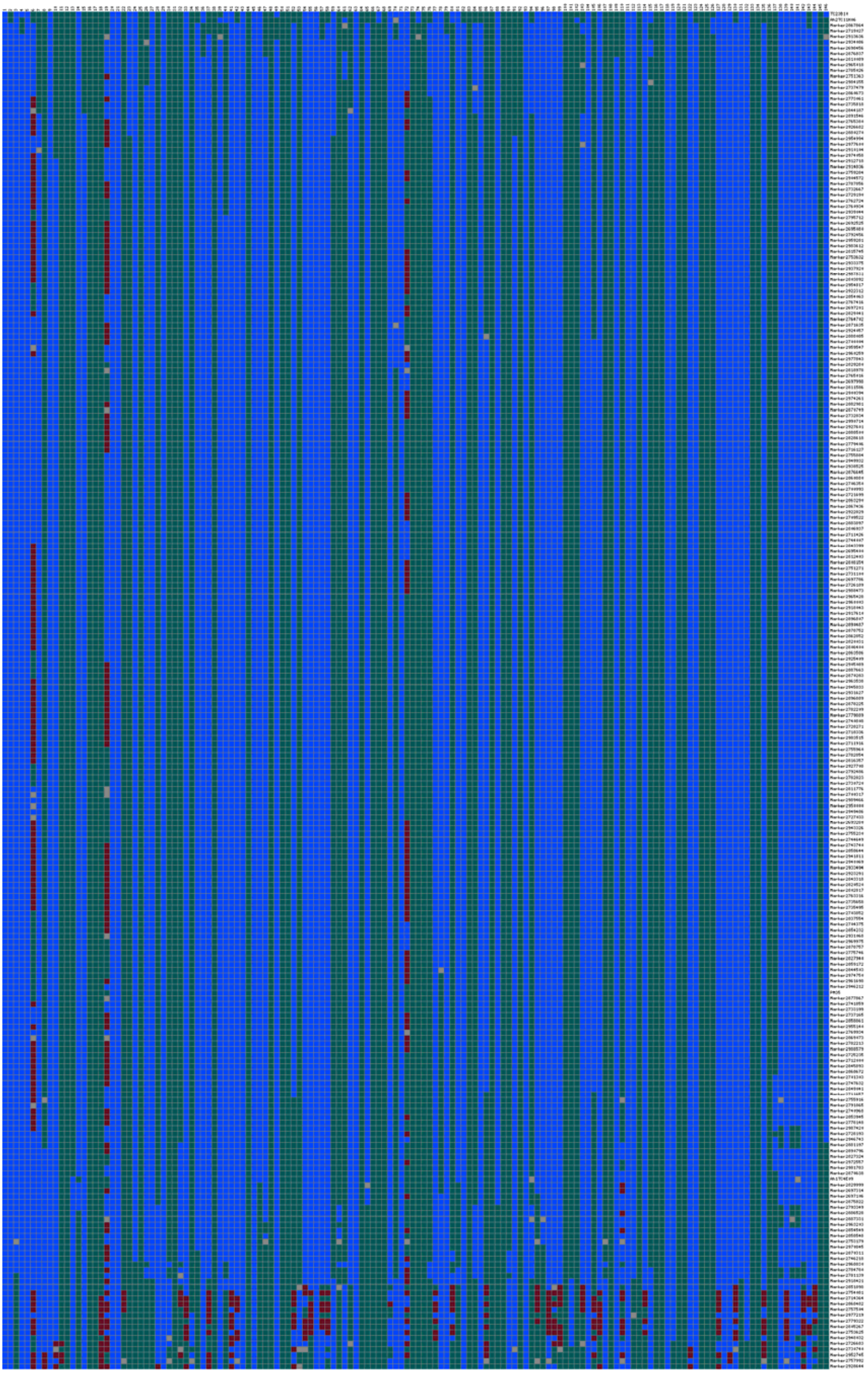

Haplotype map of the integrated map (Araip_B04)

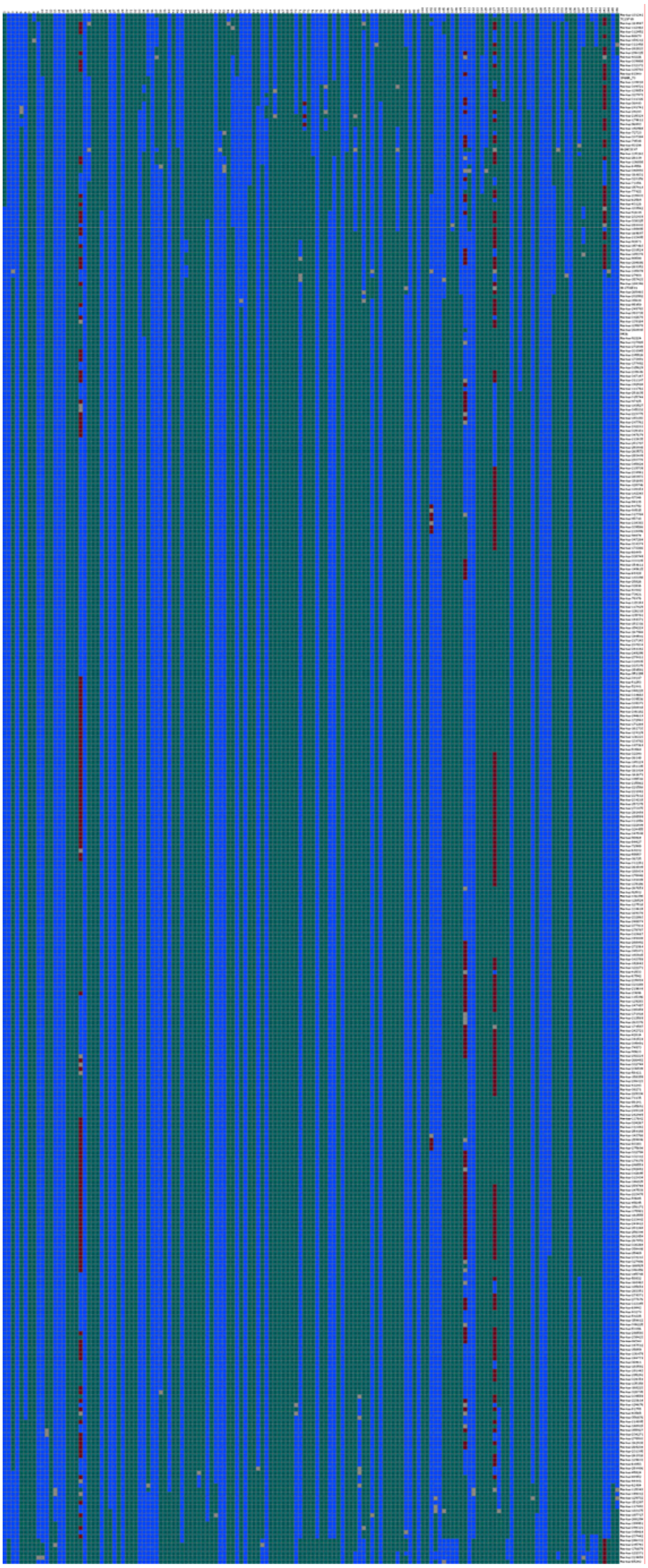

Haplotype map of the integrated map (Araip_B05)

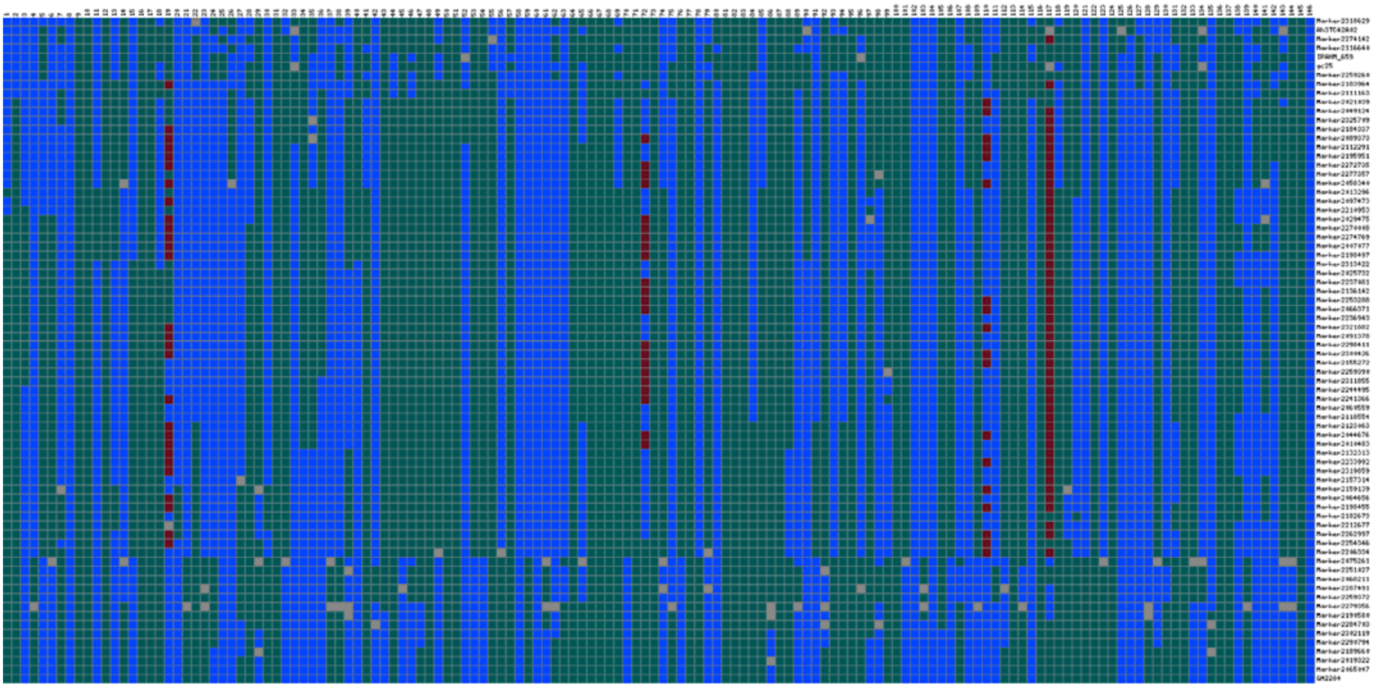

Haplotype map of the integrated map (Araip_B06)

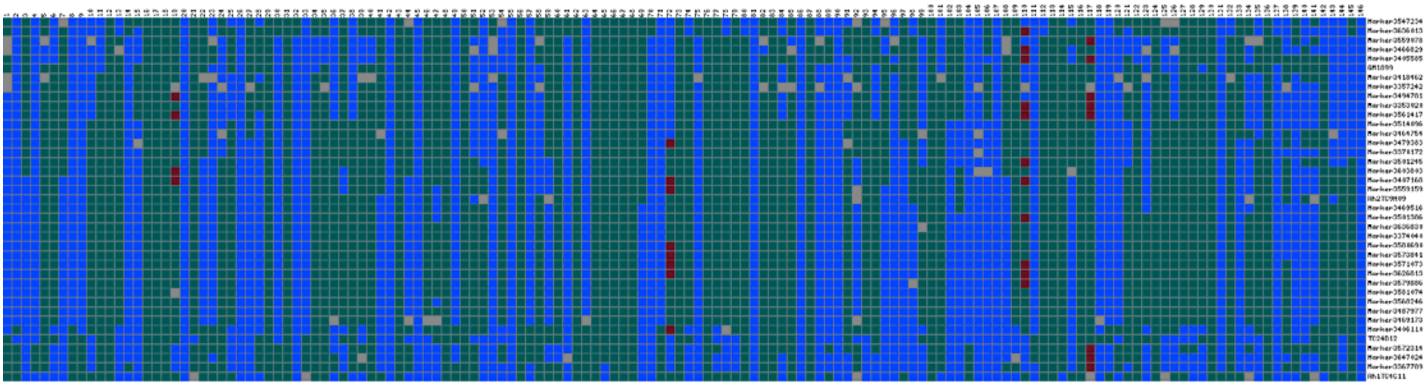

Haplotype map of the integrated map (Araip_B07)

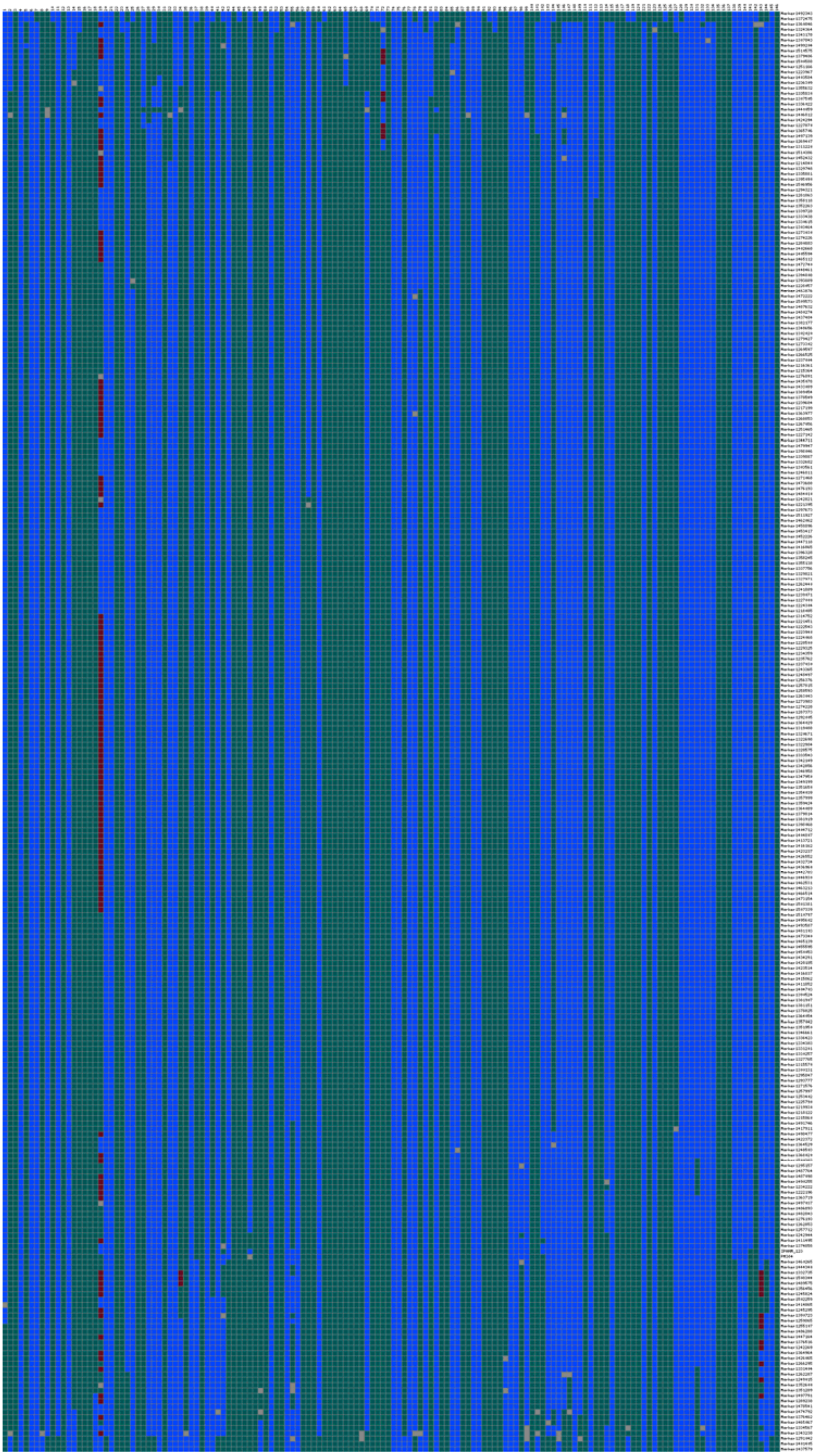

Haplotype map of the integrated map (Araip_B08)

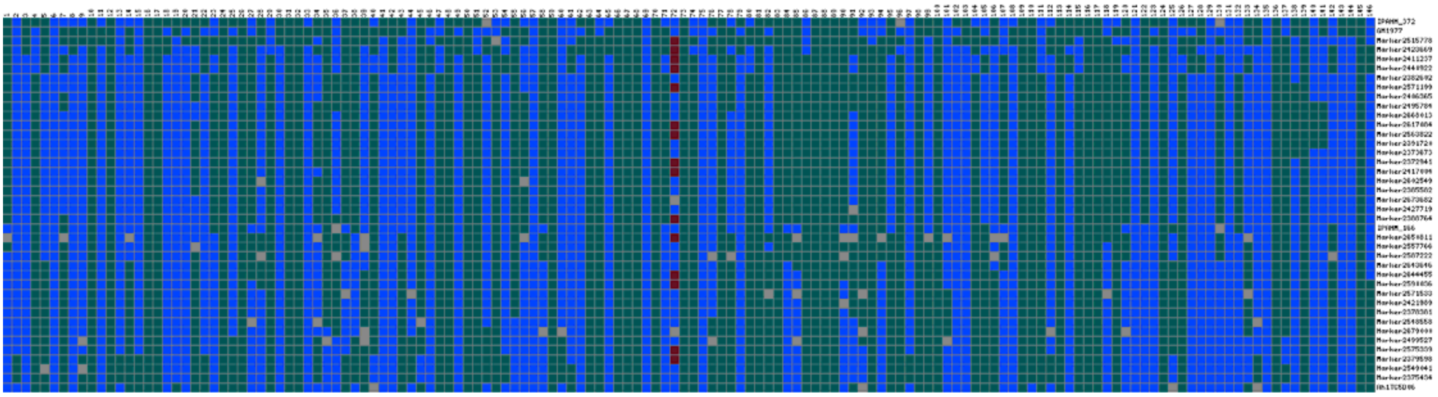

Haplotype map of the integrated map (Araip_B09)

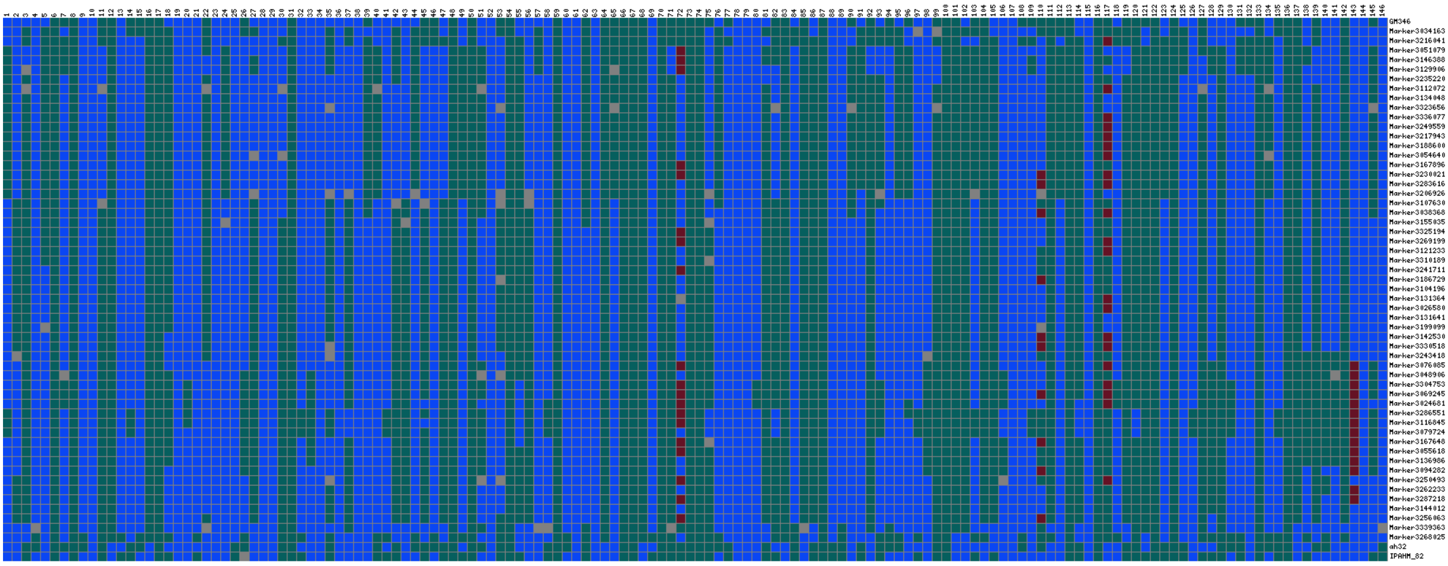

Haplotype map of the integrated map (Araip_B10)

Addition file S2 Heat map of the genetic map.

Each cell represents the recombination rate of two markers. Yellow indicates a lower recombination rate and purple a higher one.

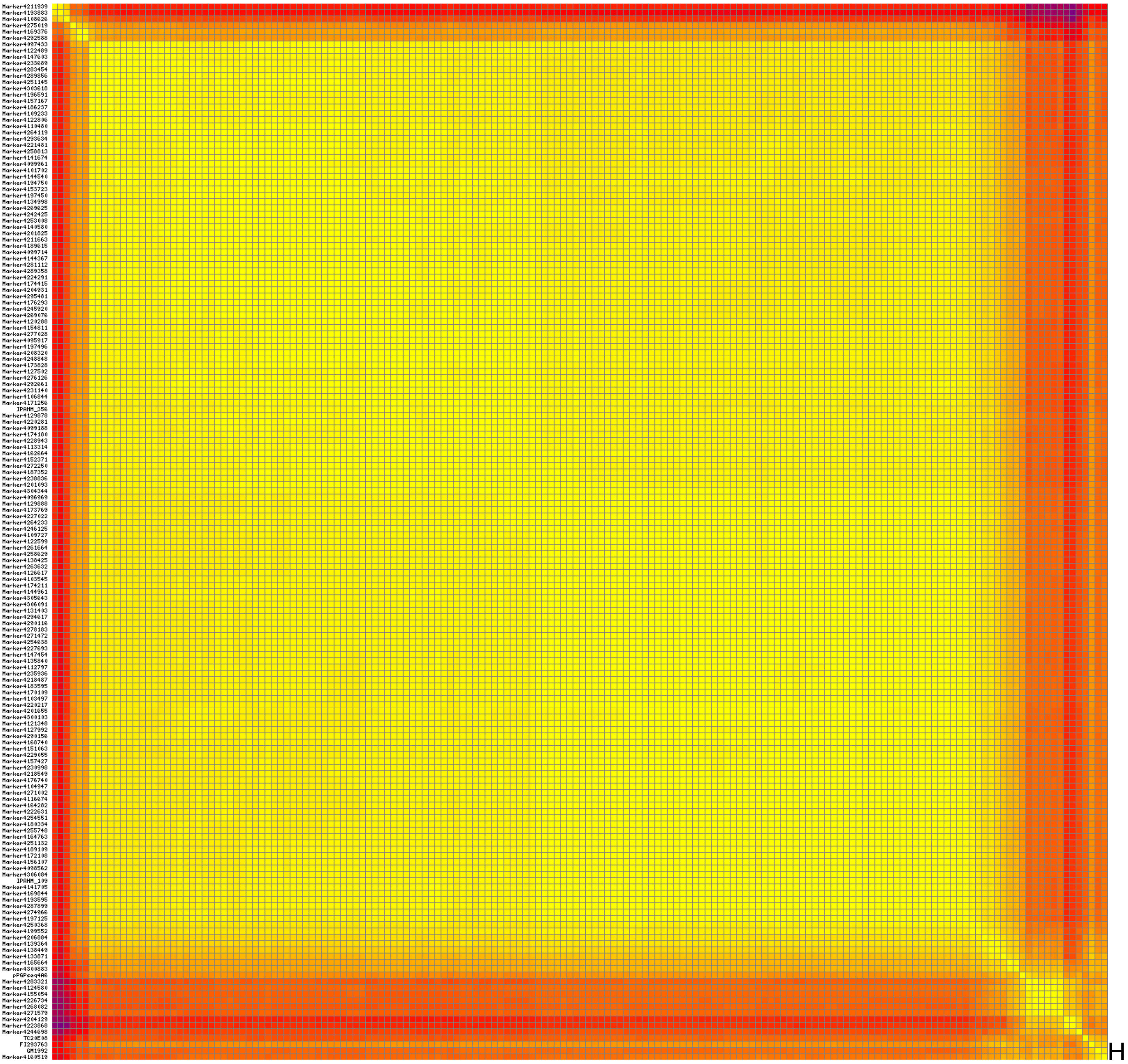

Heat map of the genetic map (Aradu_A01)

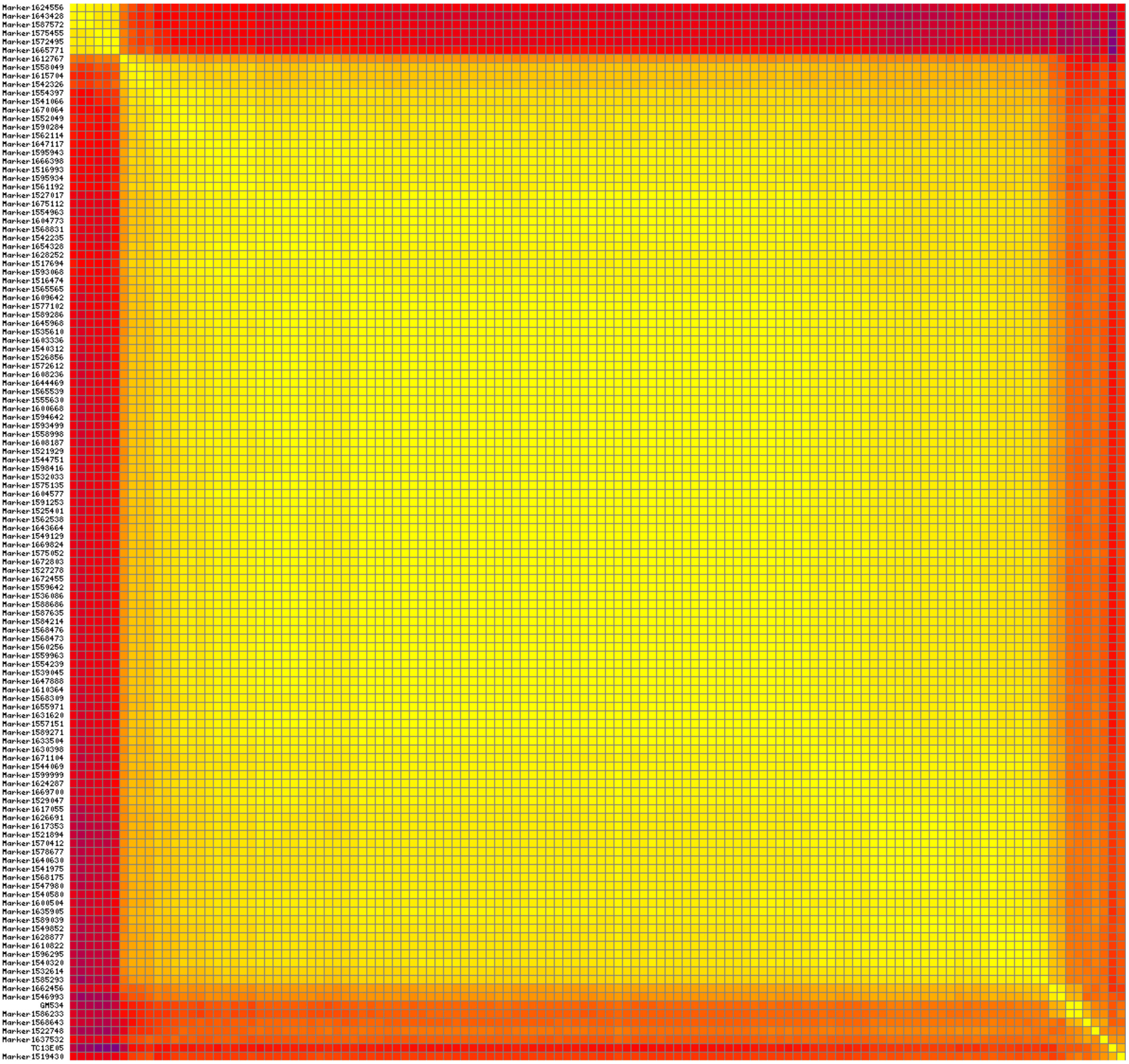

Heat map of the genetic map (Aradu_A02)

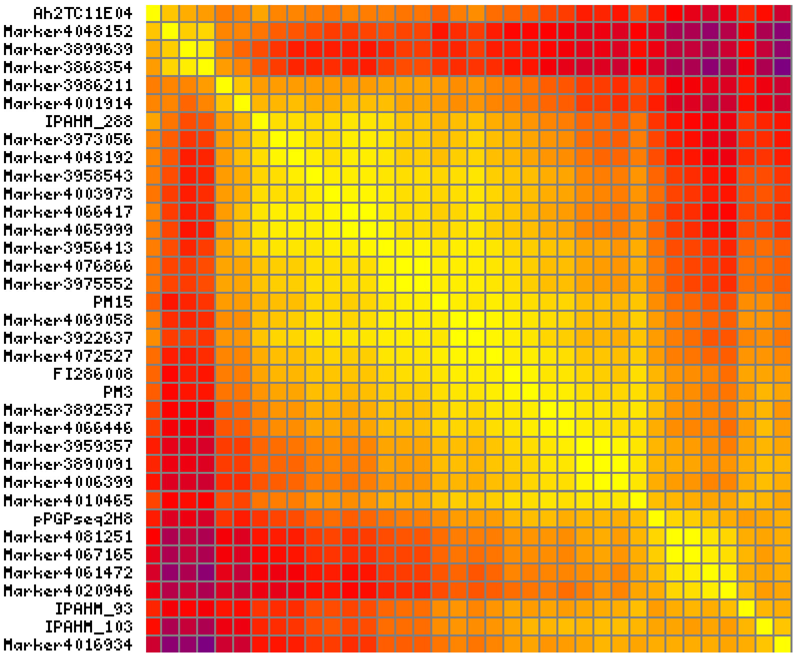

Heat map of the genetic map (Aradu_A03)

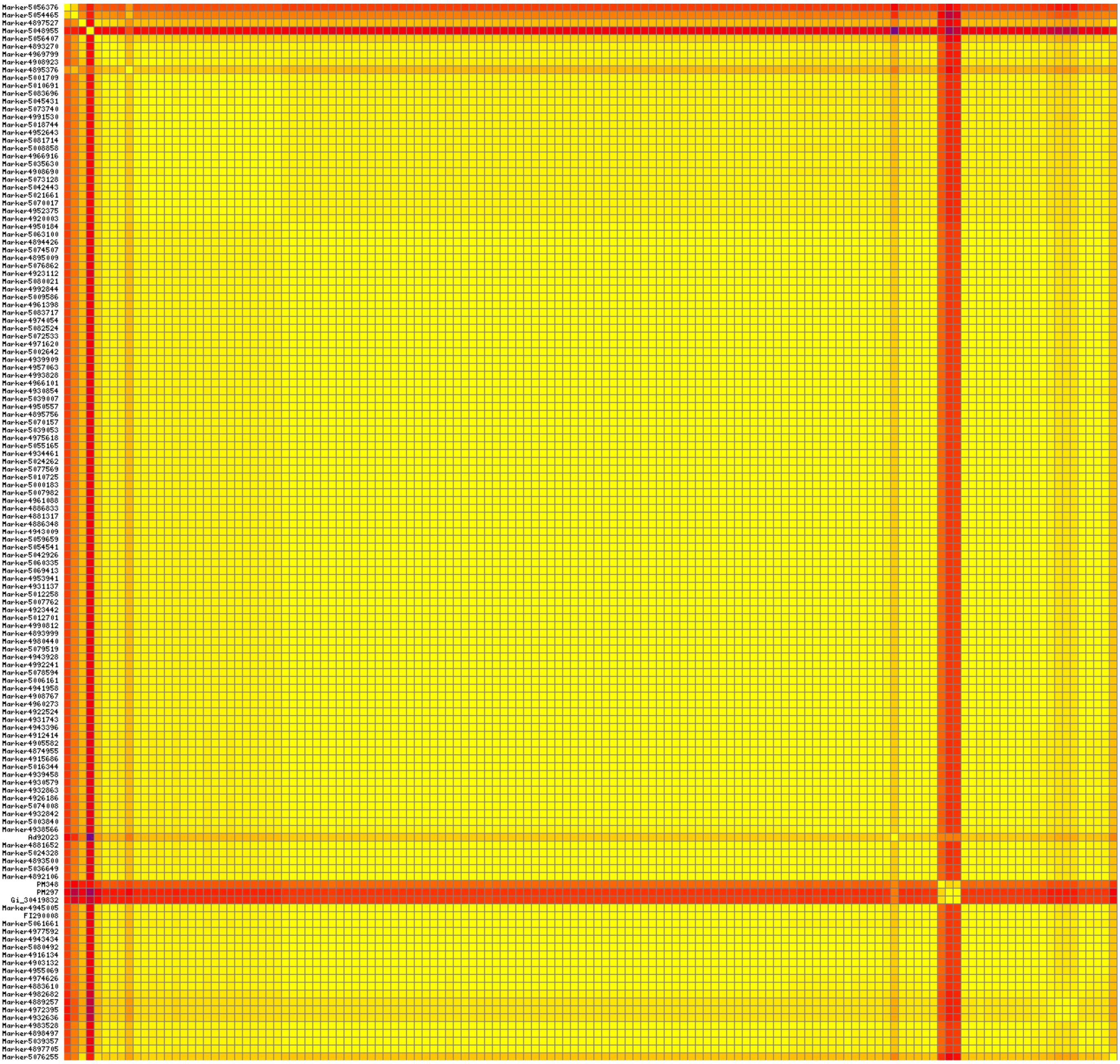

Heat map of the genetic map (Aradu_A04)

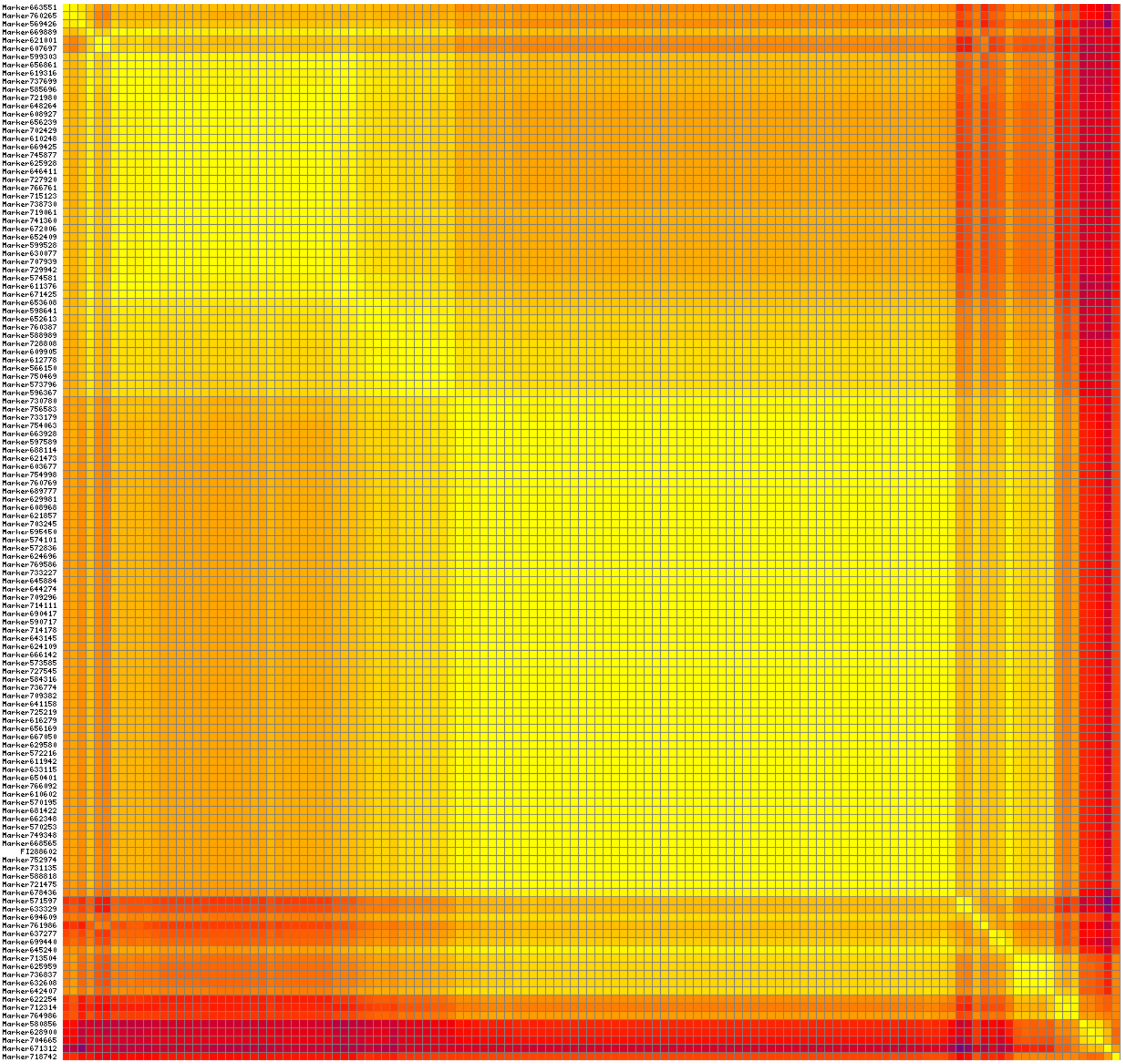

Heat map of the genetic map (Aradu_A05)

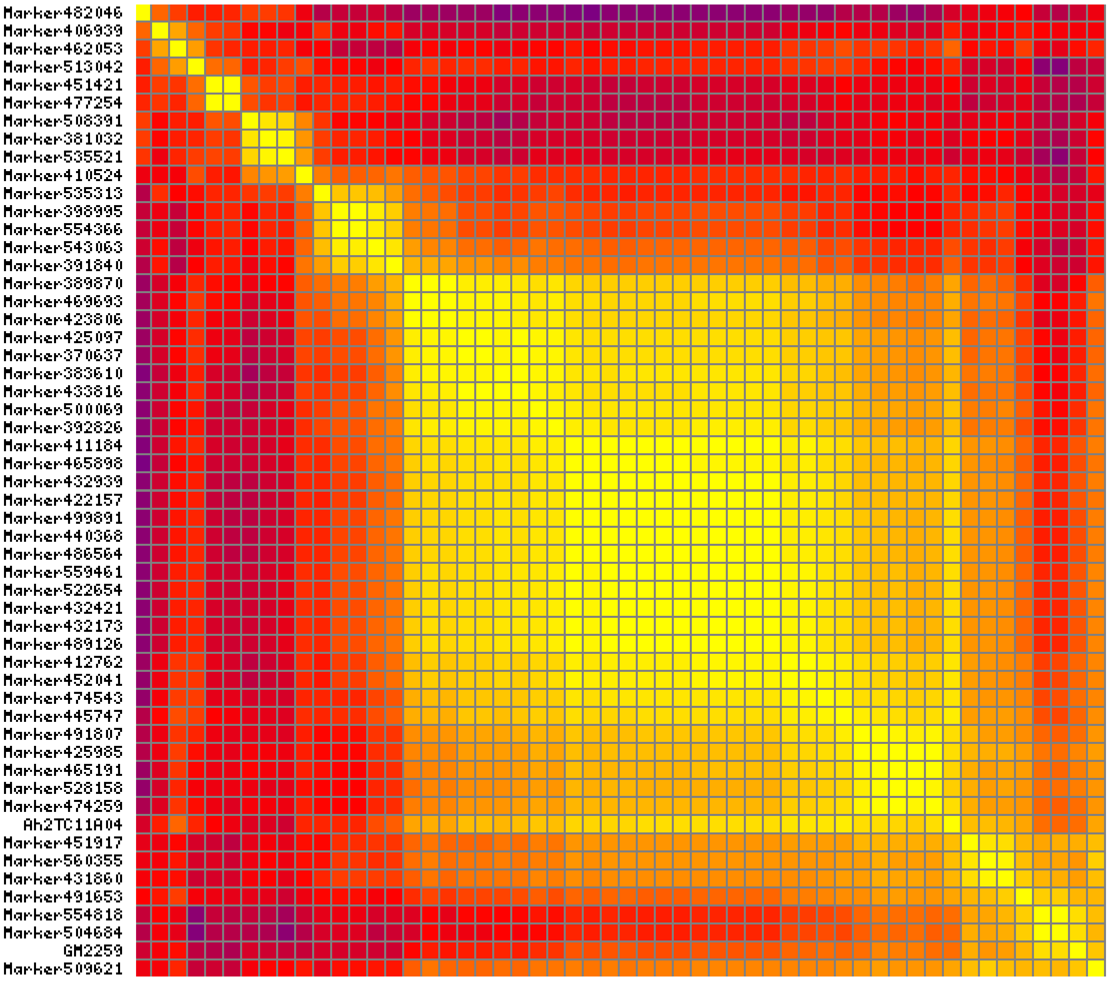

Heat map of the genetic map (Aradu_A06)

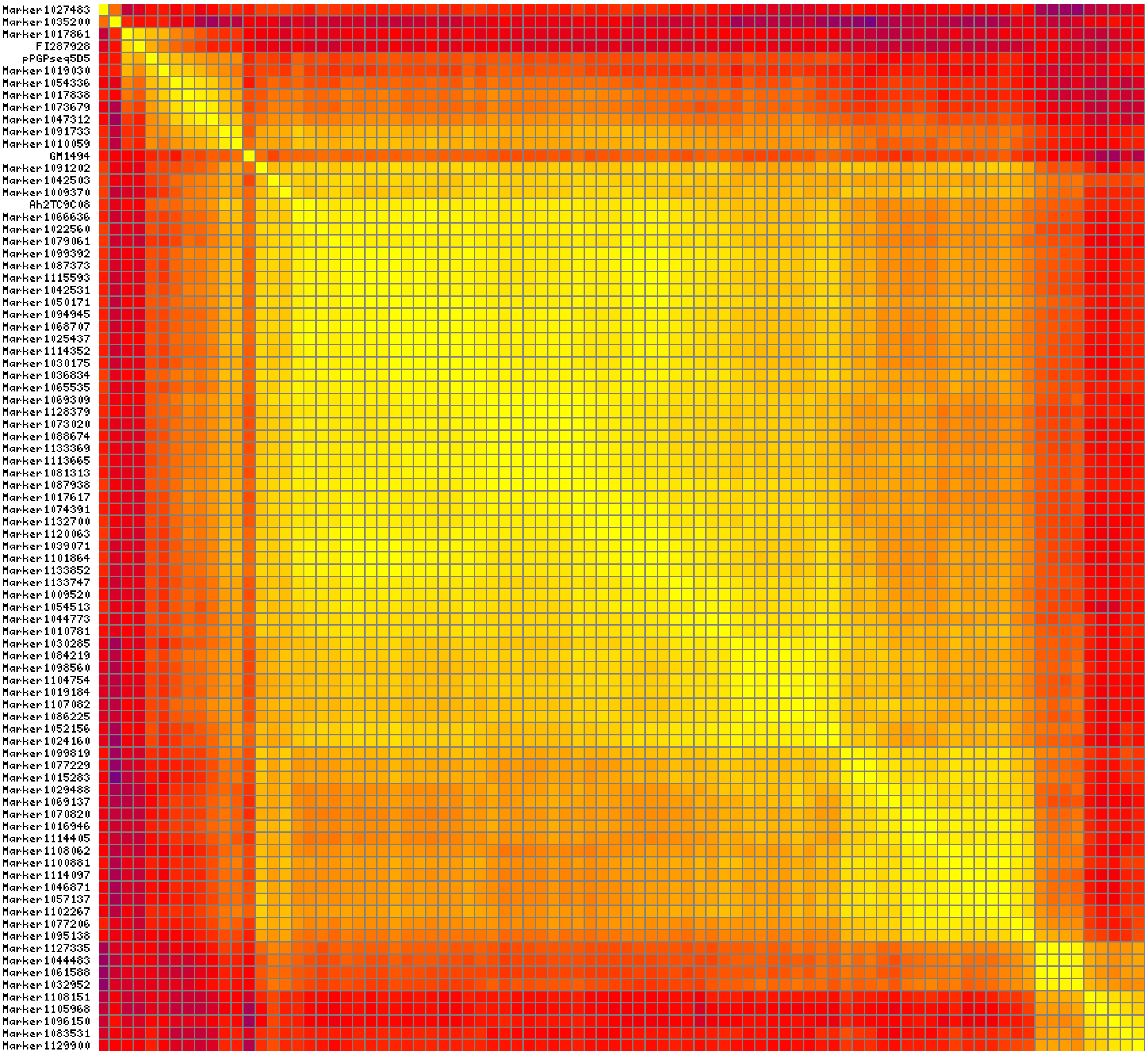

Heat map of the genetic map (Aradu_A07)

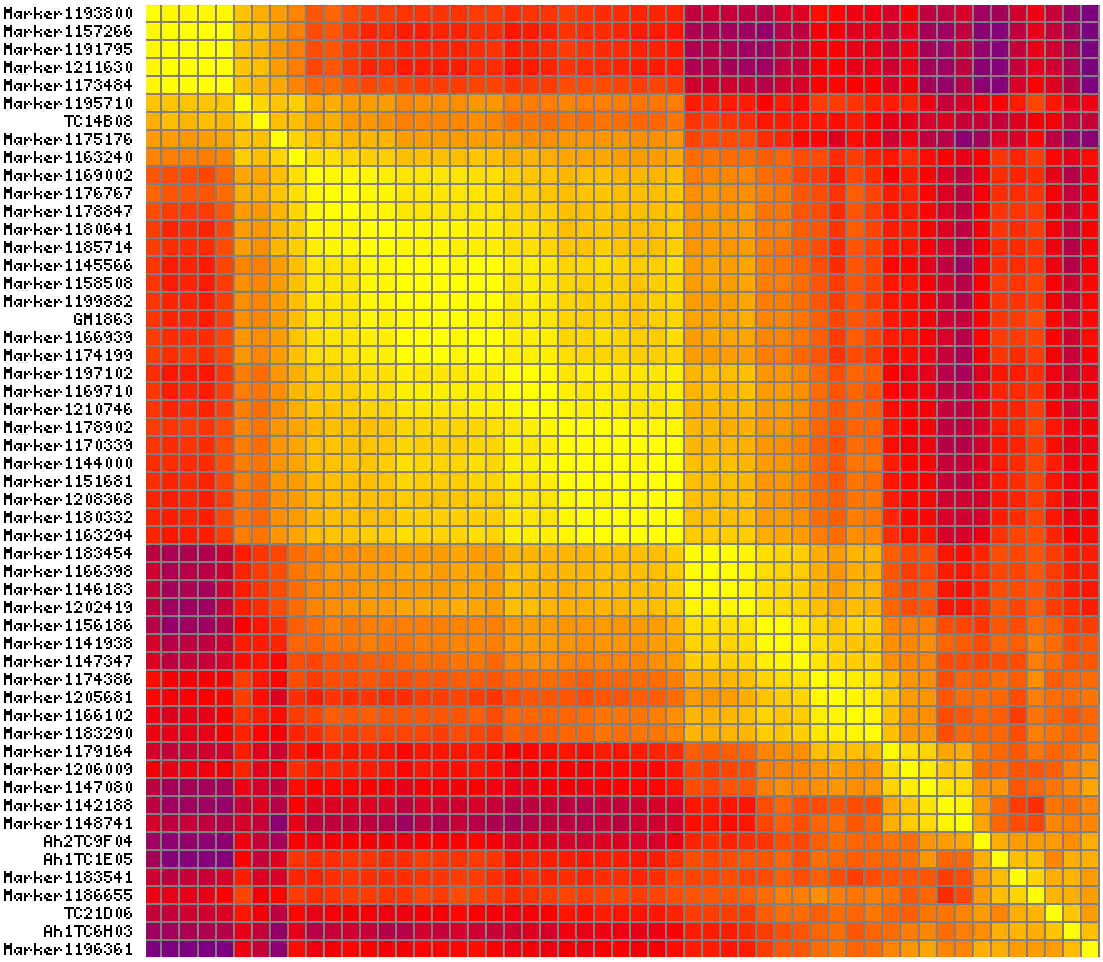

Heat map of the genetic map (Aradu_A08)

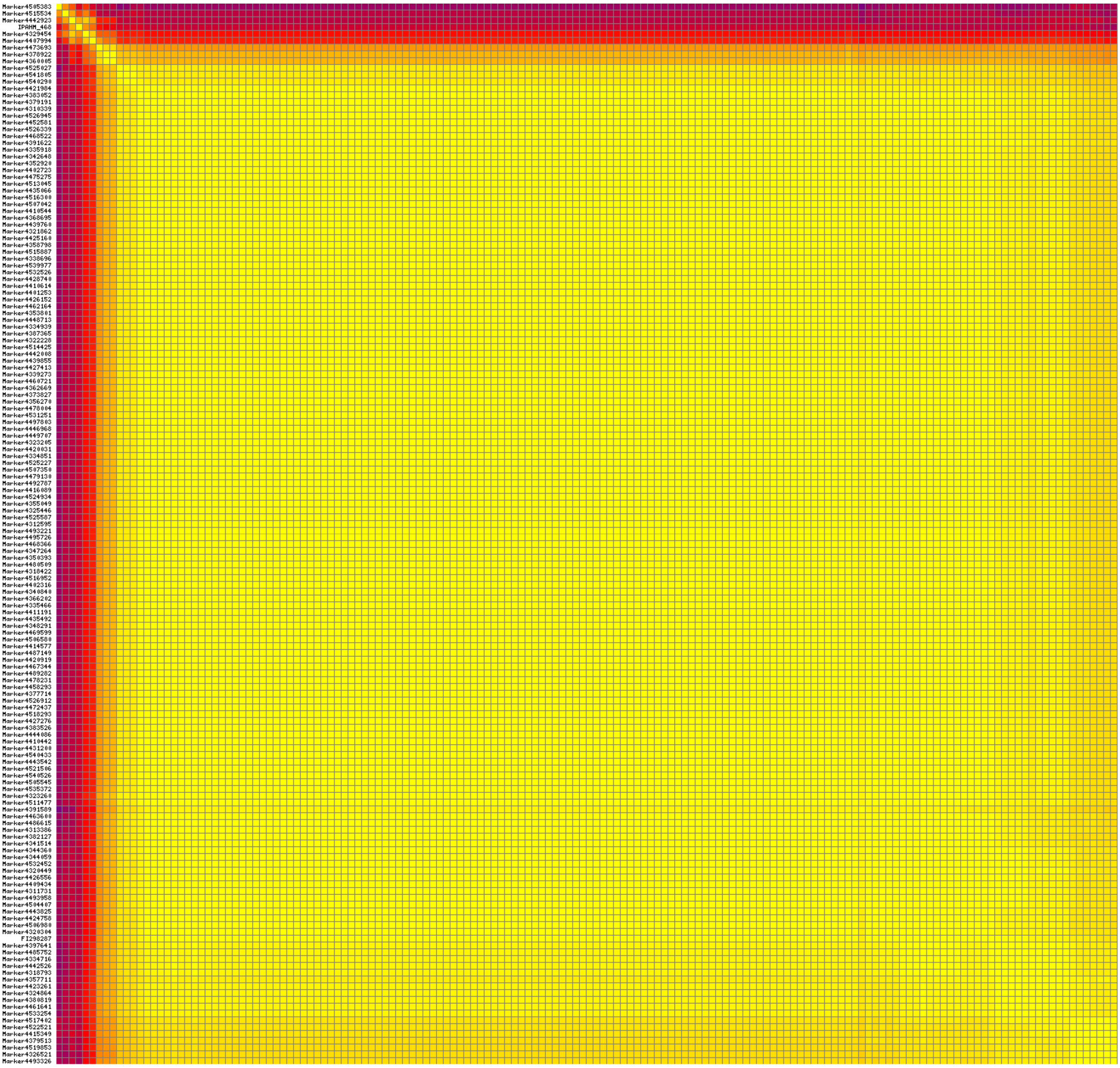

Heat map of the genetic map (Aradu_A09)

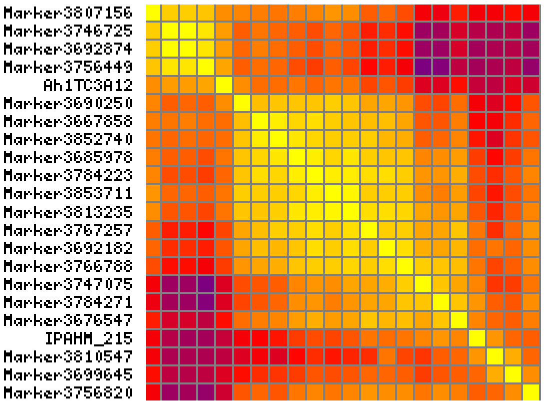

Heat map of the genetic map (Aradu_A10)

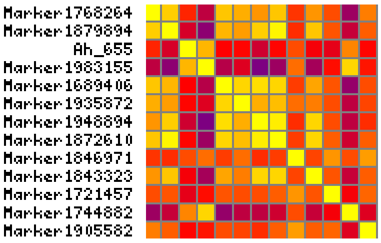

Heat map of the genetic map (Araip_B01)

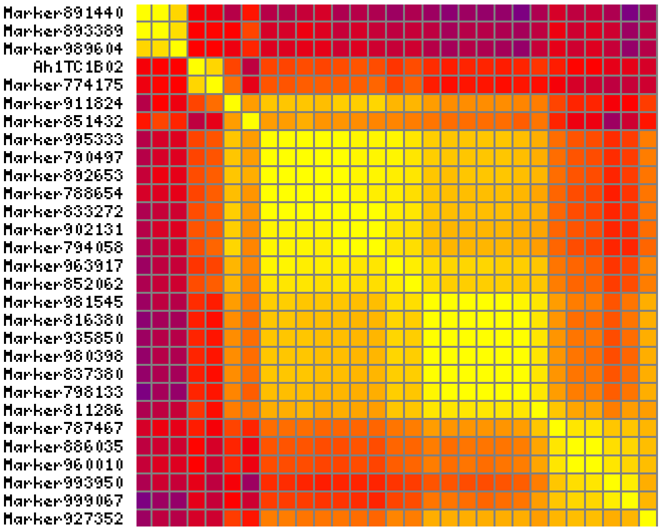

Heat map of the genetic map (Araip_B02)

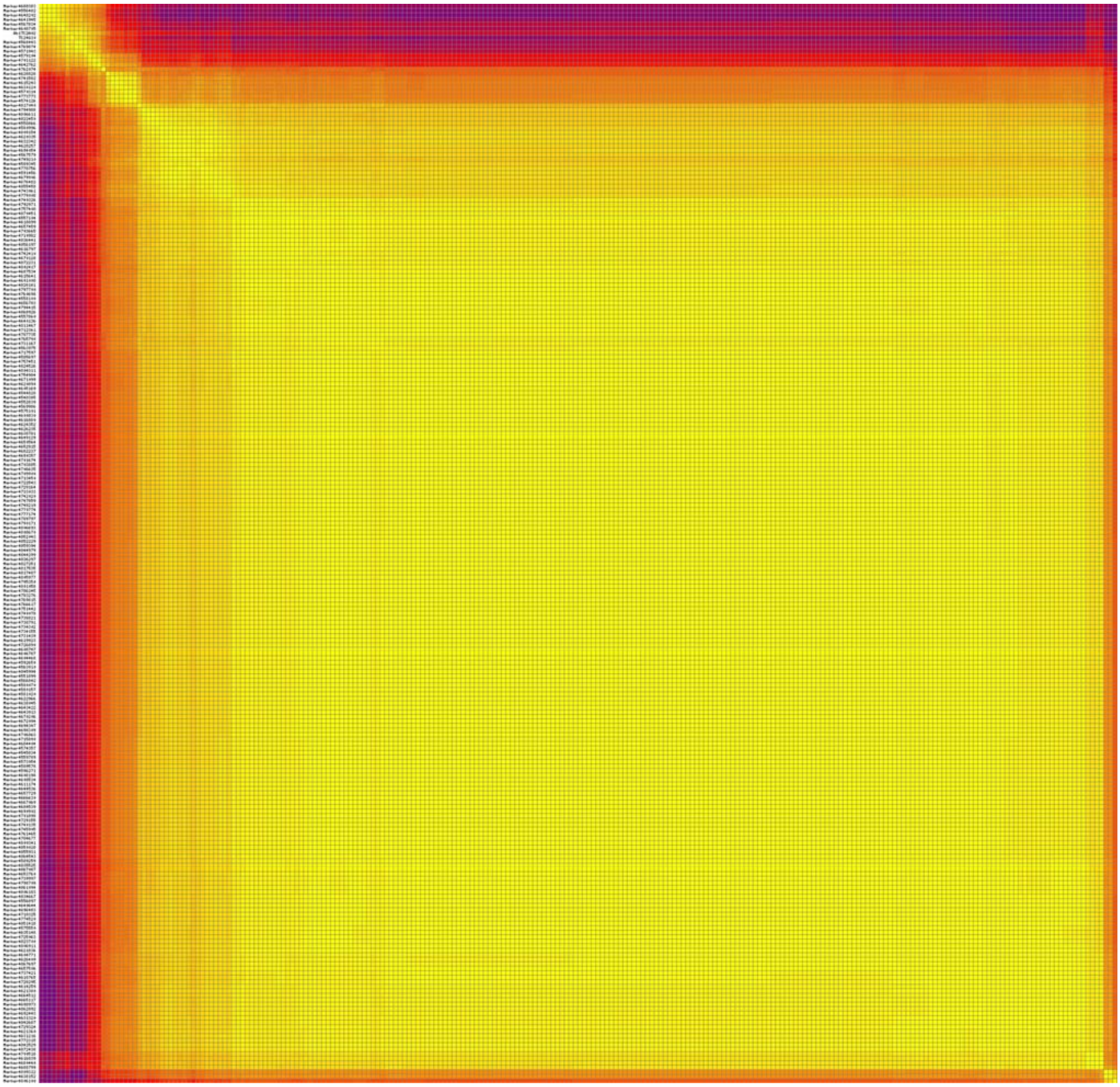

Heat map of the genetic map (Araip_B03)

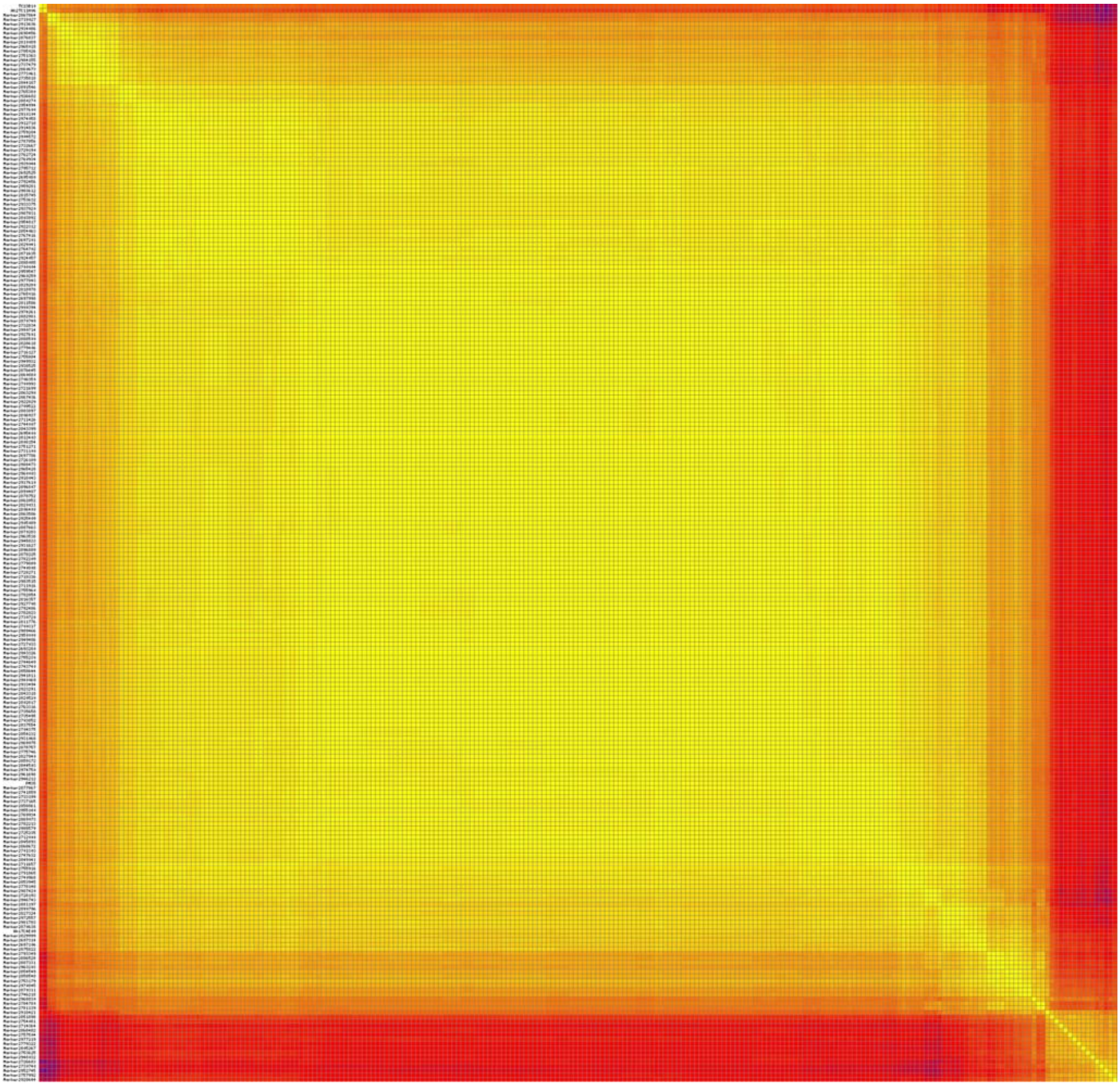

Heat map of the genetic map (Araip_B04)

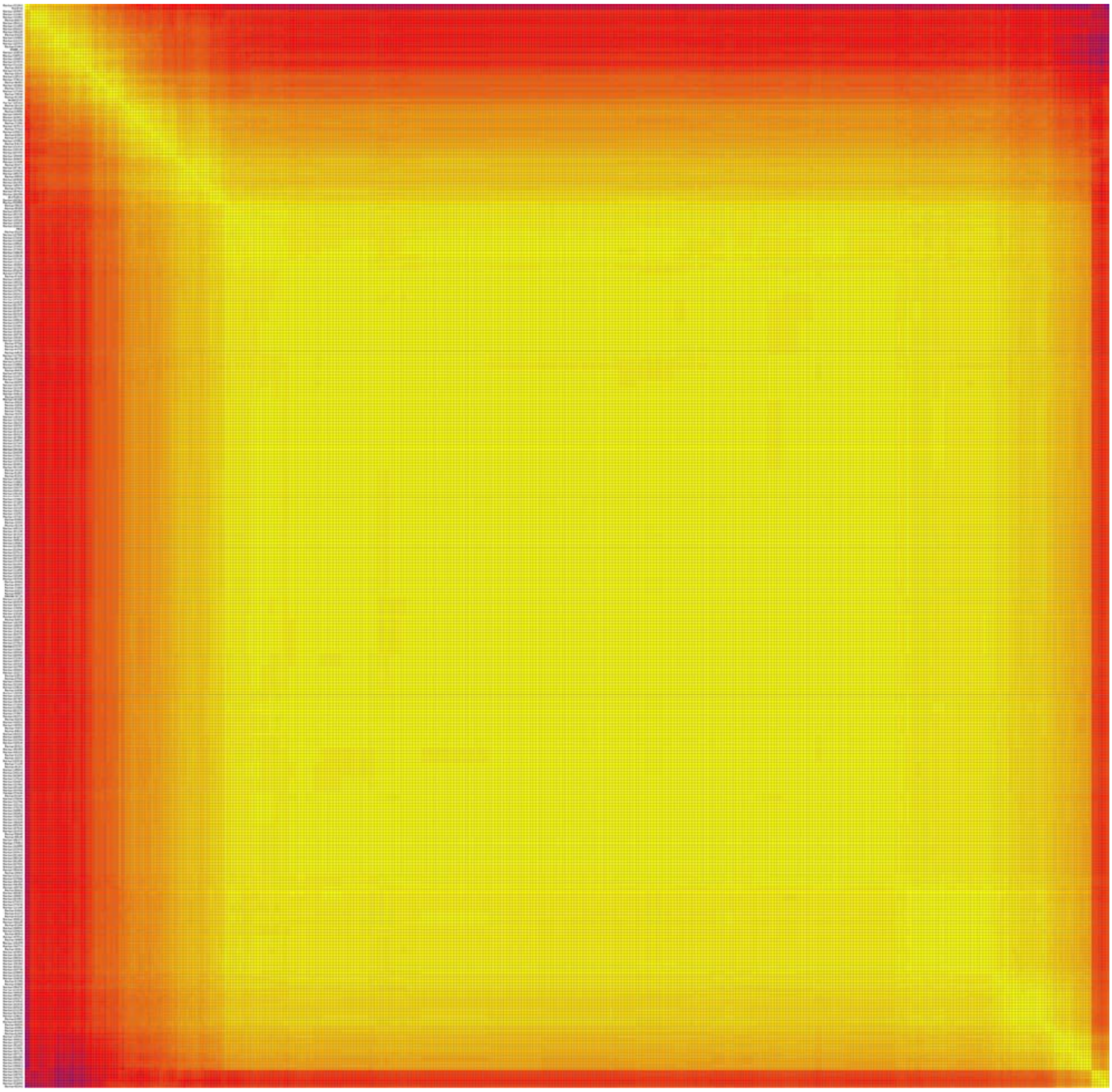

Heat map of the genetic map (Araip_B05)

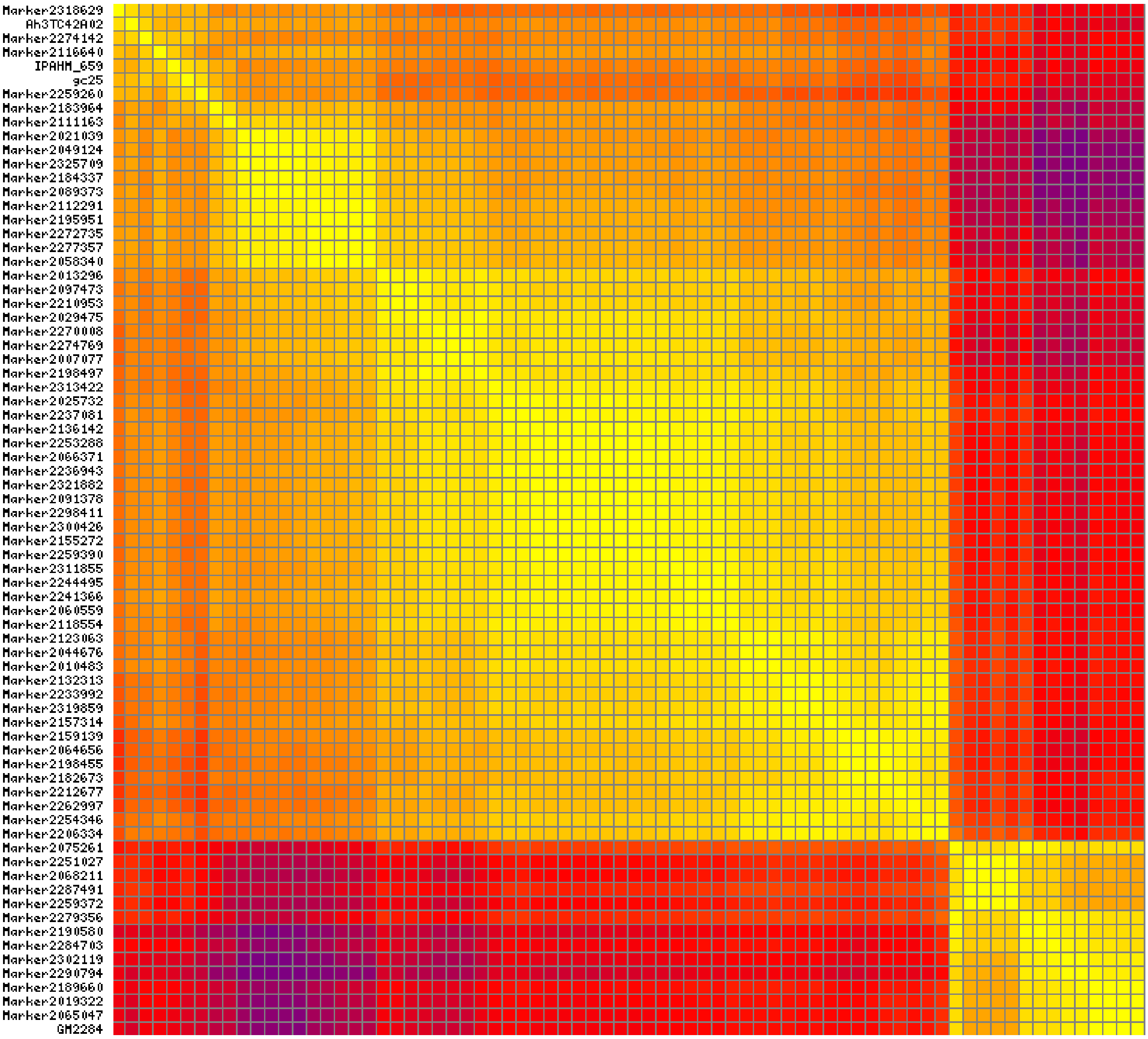

Heat map of the genetic map (Araip_B06)

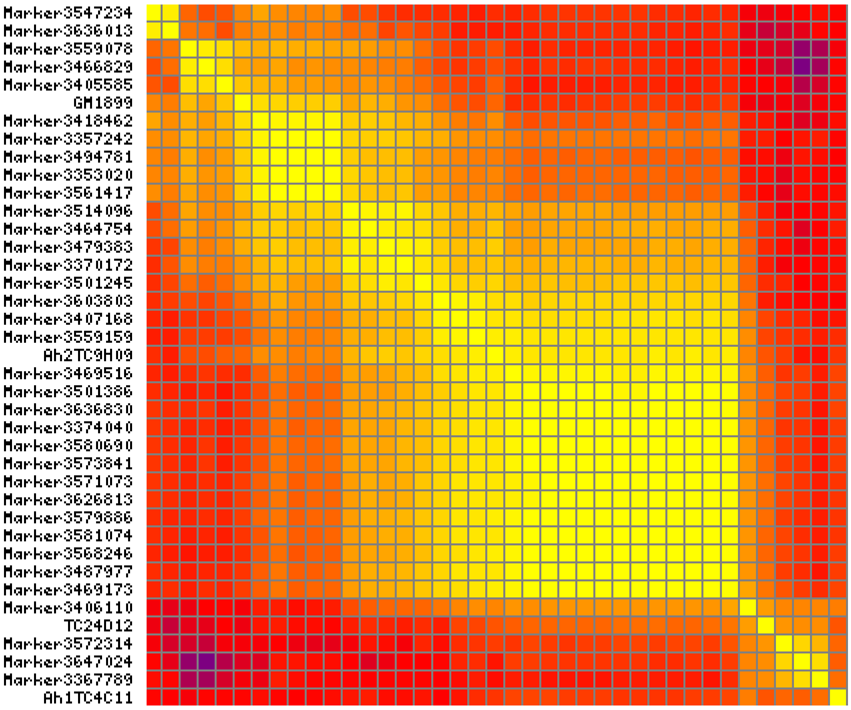

Heat map of the genetic map (Araip_B07)

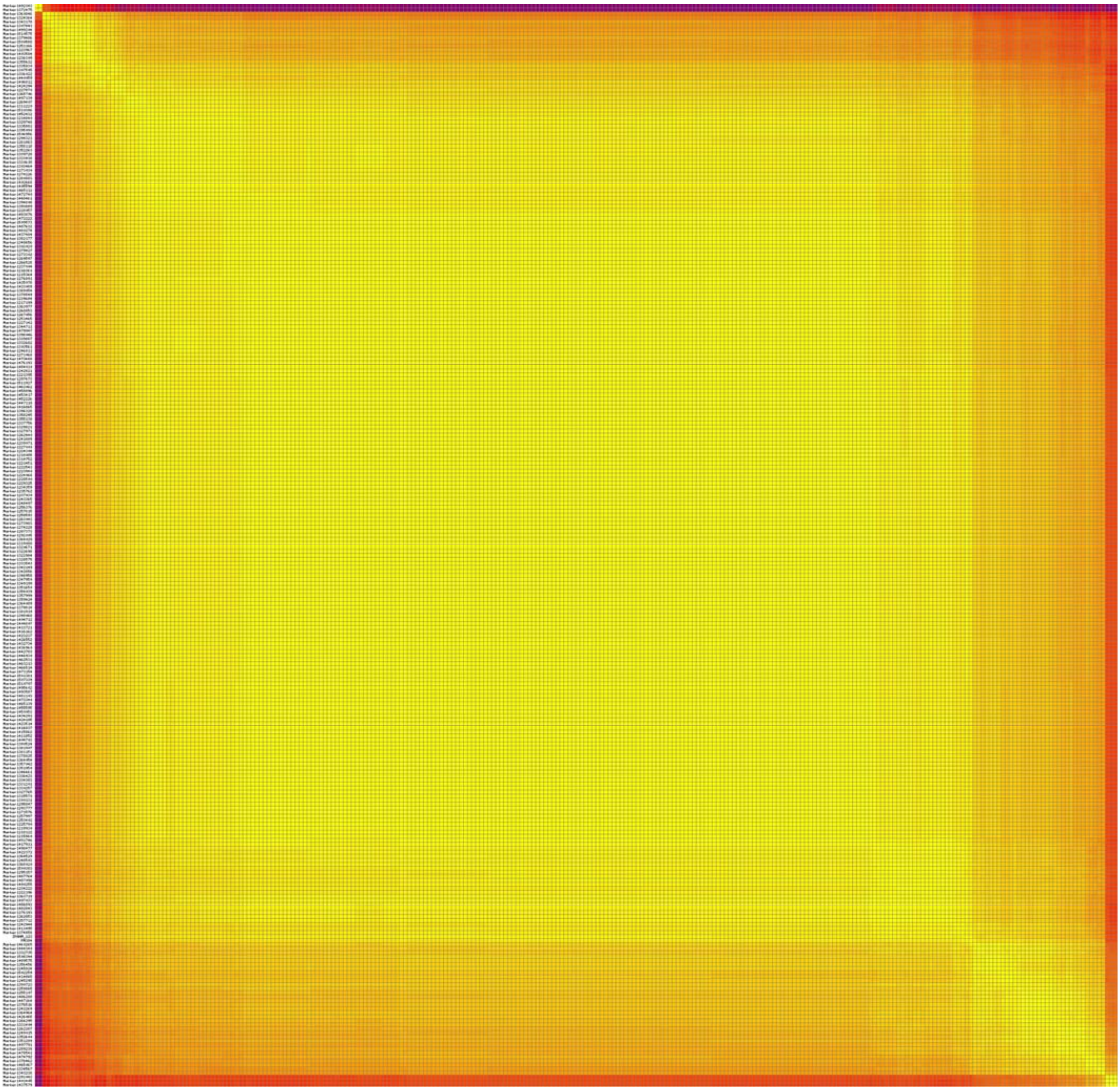

Heat map of the genetic map (Araip_B08)

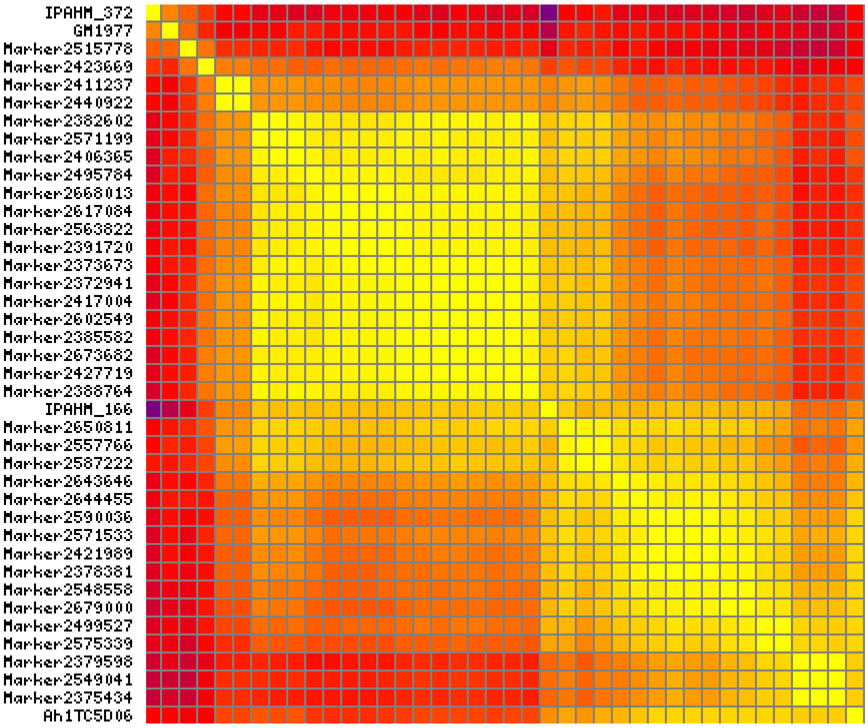

Heat map of the genetic map (Araip_B09)

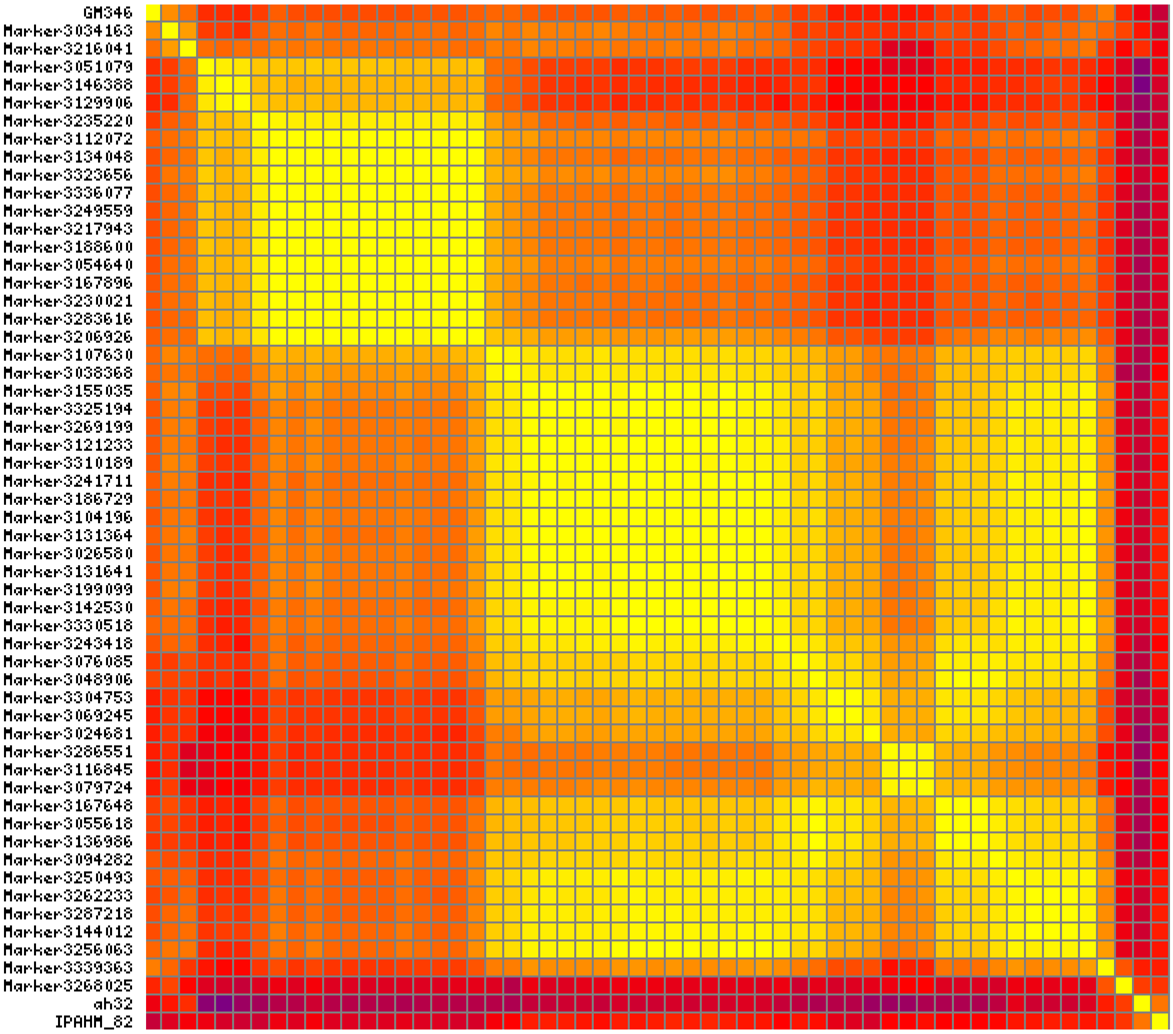

Heat map of the genetic map (Araip_B10)
